# Choice of Nanovaccine Delivery Mode Has Profound Impacts on the Intralymph Node Spatiotemporal Distribution and Immunotherapy Efficacy

**DOI:** 10.1002/advs.202001108

**Published:** 2020-08-15

**Authors:** Jianghua Wang, Shuang Wang, Tong Ye, Feng Li, Xiaoyong Gao, Yan Wang, Peng Ye, Shuang Qing, Changlong Wang, Hua Yue, Jie Wu, Wei Wei, Guanghui Ma

**Affiliations:** ^1^ State Key Laboratory of Biochemical Engineering Institute of Process Engineering Chinese Academy of Sciences 1 North 2nd Street, Zhongguancun, Haidian District Beijing 100190 P. R. China; ^2^ University of Chinese Academy of Sciences 19A Yuquan Road Beijing 100049 P. R. China

**Keywords:** delivery modes, hydrogels, lymph nodes, nanovaccines, tumor immunotherapy

## Abstract

Nanovaccines have attracted booming interests in vaccinology studies, but the profound impacts of their delivery mode on immune response remain unrealized. Herein, immunostimulatory CpG‐modified tumor‐derived nanovesicles (CNVs) are used as a nanovaccine testbed to initially evaluate the impacts of three distinct delivery modes, including mono‐pulse CNVs, staggered‐pulse CNVs, and gel‐confined CNVs. Fundamentally, delivery mode has enormous impacts on the immunomodulatory effects, altering the spatiotemporal distribution of nanovaccine residence and dendritic cell–T cell interaction in lymph nodes, and finally affecting subsequent T cell‐mediated immune performance. As a result, the gel‐confined delivery mode offers the best therapeutic performance in multiple tumor models. When extending evaluation to examine how the various delivery modes impact the performance of liposome‐based nanovaccines, similar trends in intralymph node distribution and antitumor effect are observed. This work provides a strong empirical foundation that nanovaccine researchers should position delivery mode near the top of their considerations for the experimental design, which should speed up nanovaccine development and facilitate efficient selection of appropriate delivery modes in the clinic.

## Graphical abstract

Nanovaccines constructed with mono‐pulse, staggered‐pulse, or gel‐confined delivery modehave enormous impacts on spatiotemporal distribution and immunoresponse in the lymph nodes. Comparatively, nanovaccines with gel‐confined delivery mode show pronounced lymph node accumulation and dendritic cell–T cell interaction, leading to the best antitumor immunotherapy performance in multiple tumor models. Thus, selection of an appropriate delivery mode should become an integral step for nanovaccine experimental design.

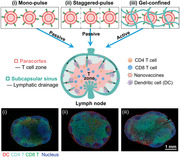

## 1 Introduction

Tumor vaccines work by stimulating tumor antigen‐specific immune response to eradicate tumor cells.^[^ ^1^ ^]^ Currently, aluminum hydroxide adjuvant has been widely used for human licensed vaccines, which acts as an antigen depot at injection site and recruits antigen‐presenting cells (APCs) for antigen uptake.^[^ ^2^, ^3^ ^]^ Inspired by this, macroscale materials‐based delivery systems (e.g., hydrogel,^[^ ^4^, ^5^, ^6^, ^7^, ^8^ ^]^ macrocapsule,^[^ ^9^, ^10^ ^]^ and microneedle^[^ ^11^, ^12^ ^]^) have been explored for modulating the recruitment, activation, and homing of APCs in situ to obtain stronger immune responses than that induced by antigen alone. With the widespread application of nanotechnologies, nanovaccines have attracted booming interests in recent years.^[^ ^13^, ^14^, ^15^ ^]^ To date, nanovaccines have been generated based on several materials, including inorganic nanoparticles (e.g., gold nanoparticles and silica nanoparticles),^[^ ^16^, ^17^, ^18^, ^19^ ^]^ organic nanoparticles (e.g., liposomes and micelles),^[^ ^20^, ^21^, ^22^, ^23^ ^]^ and even tumor‐derived vesicles (e.g., subcellular vesicles and exosomes).^[^ ^24^, ^25^ ^]^ They offer reliable protection from degradation for antigen(s), high cellular uptake into APCs, codelivery of antigen(s) and immunostimulatory molecules, as well as favorable in vivo distribution.^[^ ^26^, ^27^, ^28^ ^]^

In mammalian immune systems, lymph nodes are the primary sites wherein APCs like dendritic cells (DCs), which are indispensable for the generation of T cells response.^[^ ^14^ ^]^ Owing to their nanoscale size, nanovaccines can passively drain to lymph nodes through afferent lymphatic vessels, and majorly enter the subcapsular sinus wherein they can be taken up and processed by resident APCs (e.g., DCs).^[^ ^29^, ^30^, ^31^, ^32^ ^]^ Another area of lymph node known as the paracortex or T cell zone is the site where T cell responses are initiated via the interaction of antigen‐bearing DCs with naive T cells.^[^ ^33^ ^]^ Although nanovaccines are exceptionally efficient at achieving fast antigen entry into lymph nodes,^[^ ^34^ ^]^ the exit of some portion of a nanovaccine dose from lymph nodes via efferent lymphatic vessels results in substantial waste and precludes the maximal retention of nanovaccines in lymph nodes.^[^ ^35^, ^36^ ^]^ Given the special structure of lymph nodes, such reduced retention might further affect the distribution of nanovaccines in the lymph node areas where they can interact with DCs and ultimately initiate strong T cell responses. Thus, lymph node retention and distribution of nanovaccines should be major informative parameters in the overall immunomodulatory performance of nanovaccines.

**Scheme 1:**
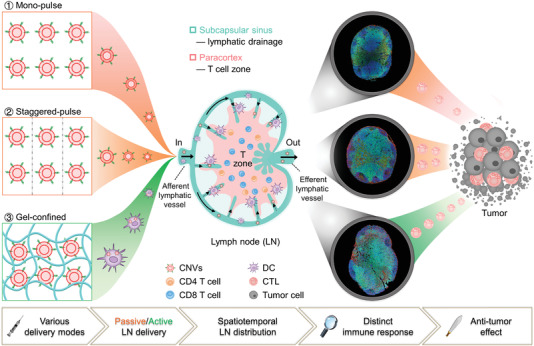
Spatiotemporal modulation of intralymph node distribution of nanovaccines for tumor immunotherapy via different delivery modes. Nanovaccines (CpG‐modified tumor‐derived nanovesicles, CNVs) were designed into three delivery modes including mono‐pulse CNVs, staggered‐pulse CNVs, and gel‐confined CNVs for tumor immunotherapy. In mono‐pulse CNVs and staggered‐pulse CNVs vaccinations, CNVs could passively drain to lymph nodes due to their nanometer size, while CNVs were actively delivered to lymph nodes through the migration of DCs under gel‐confined CNVs vaccination. Inside lymph nodes, abovementioned three CNVs delivery modes exhibited different spatiotemporal distribution of CNVs, which subsequently influenced the utilization of CNVs and the interaction of DC with T cells in T cell zone, resulting in distinct T cell‐mediated antitumor immunity.

However, such an important fact has not been realized in previous nanovaccinology studies that only use a single delivery mode.^[^ ^13^ ^]^ Inspired by this and in light of the studies showing the profound impacts of delivery mode on vaccination performance,^[^ ^37^, ^38^, ^39^, ^40^ ^]^ we initially designed different nanovaccine delivery modes to seek better spatiotemporal control over the intralymph node accumulation dynamics of nanovaccines. Briefly, we first used a single type of (tumor vesicle‐derived) nanovaccine but employed three separate delivery modes for multiple tumor vaccination applications (e.g., primary tumor, postresection recurrent tumor, and metastasis tumor) (**Scheme** **1**). Specifically, we used immunostimulatory CpG‐modified tumor‐derived nanovesicles (CNVs) to serve as a nanovaccine testbed, and evaluated single injection and multiple injection (3×) delivery modes (termed mono‐pulse and staggered‐pulse, respectively) for passive drainage into lymph nodes, as well as a hydrogel‐based antigen depot delivery mode (termed gel‐confined) to facilitate APCs‐mediated active delivery into lymph nodes. Fundamentally, our experiments clearly indicated that the delivery mode for a nanovaccine strongly impacted both the timing and spatial distribution of antigen residence in lymph nodes, and did so in a way that altered DC–T cell interaction and subsequent T cell‐mediated immunity. As a general trend, our gel‐confined delivery mode offered superior antitumor performance in all tumor models we explored. Importantly, expanding beyond a single nanovaccine material, we observed similar trends when we tested the same three delivery modes for tumor vaccination with liposome‐based nanovaccines, again demonstrating the importance of choosing a suitable delivery mode for superior antitumor immunotherapy.

## 2 Results and Discussion

### 2.1 Synthesis, Characterizations, and Immune Activation Ability of CNVs

**Figure 1:**
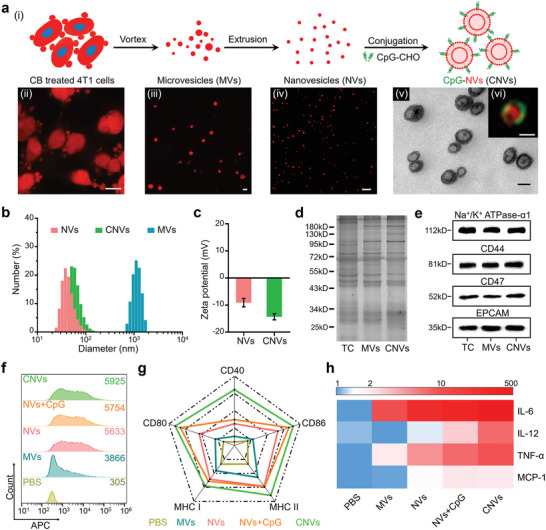
Characterizations of CNVs and their capacity for bone marrow‐derived dendritic cells (BMDCs) activation in vitro. a) Schematics for the preparation of CNVs (i), and confocal laser scanning microscope (CLSM) images of CB‐treated 4T1 cells (ii, red), released MVs (iii, red), extruded NVs (iv, red), and TEM image (v) with inserted structured‐illumination microscopy (SIM) image of CNVs (vi, CpG‐green, NVs‐red). Scale bar: (ii) 5 µm; (iii,iv) 1 µm; (v,vi) 50 nm. b,c) Size distributions and zeta potentials of MVs, NVs, and CNVs measured by DLS. d,e) SDS‐PAGE analysis for total proteins and Western blotting analysis for membrane‐associated markers (Na^+^/K^+^ ATPase‐*α*1, CD44, CD47, and EPCAM) of tumor cell (TC), MVs, and CNVs. f) Cellular uptake of MVs, NVs, NVs plus CpG, and CNVs into BMDCs. The number in the upper right corner indicated the mean fluorescence intensity (F.I.) of 5 × 10^3^ DCs. g) The expression levels of costimulatory molecules (CD40, CD80, and CD86) and MHC molecules (MHC I and MHC II) on BMDCs after 24 h incubation with MVs, NVs, NVs plus CpG, or CNVs. h) Heat map for the released cytokines levels (as fold change) in the culture supernatants of BMDCs harvested from experiment in panel (g). The data were normalized to the lowest level for each cytokine among all groups. Data in (c) represent the mean ± s.e.m. (*n* = 3).

After releasing from cytochalasin B (CB) treated 4T1 breast tumor cells via vigorous vortex, microvesicles (MVs) were then serially extruded through porous membranes (0.8, 0.2, and 0.05 µm) to obtain nanovesicles (NVs) (**Figure** **1**a i–iv). In terms of surface modification of molecules on cellular vesicle, several methods including hydrophobic interaction, chemical conjugation, and genetic engineering have been developed.^[^ ^41^, ^42^, ^43^, ^44^, ^45^, ^46^, ^47^ ^]^ Considering the efficacy and stability of CpG ODN modification, we herein chose a covalent conjugation process to modify the extruded NVs. Specifically, we ordered 26‐base CpG with an aldehyde group (‐CHO) at its 5′ end. Such a custom synthesis enabled direct conjugation of CpG onto NVs through covalent reaction between CpG's CHO group and membrane protein's amino group. Transmission electron microscopy (TEM) analysis showed that the resultant CNVs had an average diameter of 30–100 nm and had their round vesicular structure (Figure 1a v). We also synthesized a CpG variant with a fluorophore at its 3′ end, and green fluorescence was observed around NVs (red) (Figure 1a vi), indicating the successful preparation of the CNVs. Moreover, the dynamic light scattering (DLS) analysis revealed that the CNVs had slightly increased diameters and slightly decreased zeta potential compared to unconjugated NVs, findings consistent with the surface modification of the negative charge bearing CpG (Figure 1b,c). Sodium dodecyl sulfate polyacrylamide gel electrophoresis (SDS‐PAGE) and immunoblotting analyses of membrane protein in tumor cell and CNVs confirmed the retention of similar global protein populations as well as multiple tumor‐cell‐specific markers like CD44, CD47, and EPCAM (Figure 1d,e).

Having confirmed the antigen and CpG coloading capacities of the CNVs, we next investigated whether they had immune activation ability for DCs in vitro. Indeed, the greatest extent of DC uptake was observed for the conjugated CNVs, and it was notable that all of the nanoscale materials had significantly improved uptake compared to the microscale vesicles (Figure 1f). After internalization, CpG could be released in the lysosome for the reaction with the toll‐like receptor 9 to promote the DC activation, and the NVs loaded with natural tumor antigen could be utilized for presentation (Figure S1, Supporting Information). As a result, the levels of costimulatory markers (CD40, CD80, and CD86) and major histocompatibility complex (MHC) molecules (MHC I and MHC II) (Figure 1g; Figure S2a, Supporting Information), as well as secretory cytokines levels (IL‐6, IL‐12, TNF‐*α*, and MCP‐1), were highest for the DCs incubated with CNVs (Figure 1h; Figure S2b, Supporting Information). Collectively, these results established that our conjugated CNVs were appropriately sized and had sufficient DC activation capacity, which made them suitable for serving as a nanovaccine testbed in comparison of multiple delivery modes.

### 2.2 Recruitment Behavior of Gel‐Confined CNVs In Vivo

**Figure 2:**
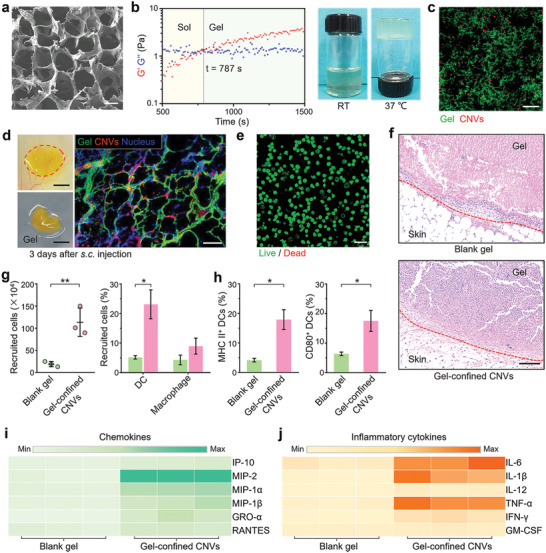
Characterizations of gel‐confined CNVs and their capacity for DCs recruitment and activation in vivo. a) Scanning electron microscopy (SEM) image of blank hydrogel (blank gel). Scale bar: 25 µm. b) The evolution of dynamic storage modulus (*G*′) and loss modulus (*G*″) of gel‐confined CNVs at 37 °C (left), and photographs of gel‐confined CNVs before and after incubation at 37 °C (right). c) CLSM image of gel (green)‐confined CNVs (red). Scale bar: 25 µm. d) Photographs of the retrieved skin and gel sample (left) from a mouse at day 3 post‐s.c. injection with gel‐confined CNVs, and the frozen‐section fluorescent image (right) of the retrieved gel sample. Scale bar: left, 5 mm; right 25 µm. e) Live (green)/Dead (red) stained image of isolated cells from the retrieved gel sample. Scale bar: 25 µm. f) H&E stained images of skin samples from mice of the blank gel and gel‐confined CNVs groups at day 3 post‐s.c. injection. Scale bar: 100 µm. g) Quantitation of recruited cells (left) within the retrieved gels at day 3 post‐s.c. injection and the corresponding proportions of DC and macrophage (right). h) Proportions of CD80^+^ CD11c^+^ and MHC II^+^ CD11c^+^ cells in recruited cells. i,j) Heat map for the concentrations of chemokines and inflammatory cytokines in lysates of the isolated cells from retrieved gels at day 3 postinjection. Data in (g) and (h) represent the mean ± s.e.m. (*n* = 3). *P*‐values between two groups were calculated via unpaired Student's *t*‐test. *P < 0.05, **P < 0.01.

In addition to passive lymph node draining, another approach for delivering CNVs to lymph nodes is active delivery by APCs like DCs.^[^ ^48^, ^49^ ^]^ Recent studies have revealed that scaffolds made of various materials can be used to retain antigens at injection site, where antigens can be recognized and taken up into recruited APCs then subsequently delivered to lymph nodes by APCs.^[^ ^4^, ^50^, ^51^ ^]^ We envisioned that we could potentially use hydrogel to retain the CNVs at the vaccination site, thereby achieving APCs‐mediated active lymph nodes delivery of CNVs. Pursuing this, we developed a method for embedding the CNVs into a thermosensitive hydrogel as an antigen depot at the injection site.^[^ ^52^, ^53^ ^]^ Considering biocompatibility and almost nonadjuvant property (Figure S3, Supporting Information), we chose chitosan glutamate (CG) to prepare a thermosensitive hydrogel through physical crosslinking with *α*, *β*‐glycerophosphate, which existed as a solution with low viscosity at room temperature (RT) but transited into a gel at 37 °C (Figure S4, Supporting Information). We anticipated that we could gently mix CNVs with the liquid form of the hydrogel and then the body temperature of mice would facilitate formation of a gel (postvaccination), thereby embedding CNVs in its porous network (**Figure** **2**a; Figure S4, Supporting Information) to form what we termed gel‐confined CNVs.

We mixed the CNVs and the hydrogel solution at room temperature in a 3:7 volume ratio, and noted that the inclusion of CNVs did not greatly affect the rheological behavior of hydrogel, causing only a slight increase for the gelation time (Figure 2b; Figure S4, Supporting Information). Subsequently, staining demonstrated that the CNVs could be successfully embedded in the porous cavities of the formed hydrogel (Figure 2c). Further, we found that subcutaneous (s.c.) injection into mice led to formation of a CNVs depot at the injection site and did not cause any obvious skin damage or other deleterious effects (Figure 2d; Figure S5a, Supporting Information). The hydrogel gradually degraded within three weeks after injection (Figure S5a,b, Supporting Information), and analysis of sections prepared from a retrieved gel at day 3 postinjection revealed clear infiltration of viable cells into the hydrogel (Figure 2e), and such phenomenon was still observed two weeks after injection (Figure S5c, Supporting Information).

We next conducted experiments in which blank hydrogel or gel‐confined CNVs were applied to the backs of mice. Hematoxylin and eosin (H&E) staining of tissue sections retrieved from the injection site at day 3 postinjection revealed significantly increased cellular infiltration in the gel‐confined CNVs group compared to the blank hydrogel sample (Figure 2f). To characterize the nature of the cells recruited into the gels, we gently homogenized recovered gel material and used flow cytometry to assess cell types and cytokine expression. Compared to blank hydrogel, embedding of the CNVs resulted a significant increase in cell recruitment into gel‐confined CNVs sample, and flow cytometry further demonstrated that DCs were the major cells among recruited APCs, which also accompanied with a few macrophages (Figure 2g). Such substantial increase in APCs recruitment was attributed to significantly increased chemokines production (Figure 2i; Figure S6a, Supporting Information). Moreover, the significantly increased inflammatory cytokines levels we detected in the gel‐confined CNVs samples could help explain the increased levels of DC activation assessed as the proportion of CD80^+^ CD11c^+^ and MHC II^+^ CD11c^+^ cells (Figure 2h,j; Figure S6b, Supporting Information). Collectively, these results demonstrated successful development of a suitable hydrogel‐based depot delivery mode (gel‐confined CNVs) to facilitate inclusion of APCs‐mediated active delivery of CNVs in our comparison of the multiple delivery modes for CNVs.

### 2.3 Spatiotemporal Intralymph Node Distribution of CNVs and Immune Cells under Three Delivery Modes

Having established suitable immunogenic materials with the CNVs and a hydrogel‐based delivery system to permit comparison of passive draining versus APCs‐mediated active delivery of CNVs into lymph nodes, we were able to conduct experiments to rigorously explore the vaccination‐performance‐related characteristics of three distinct delivery modes. Note that all three of the delivery modes we tested used the same total amount of CNVs. For passive delivery, we tested two modes seeking to investigate whether extending the lymph node draining time window might affect the extent or nature of immune responses: a single injection of high dose formulation of CNVs (termed mono‐pulse CNVs) and multiple injection (3×) of low doses once every 2 d (termed staggered‐pulse CNVs). Moreover, the inclusion of gel‐depot (termed gel‐confined CNVs) enabled us to experimentally dissect the specific lymph node distribution pattern and immune elicitation consequences of passive versus active nanovaccine delivery.

**Figure 3:**
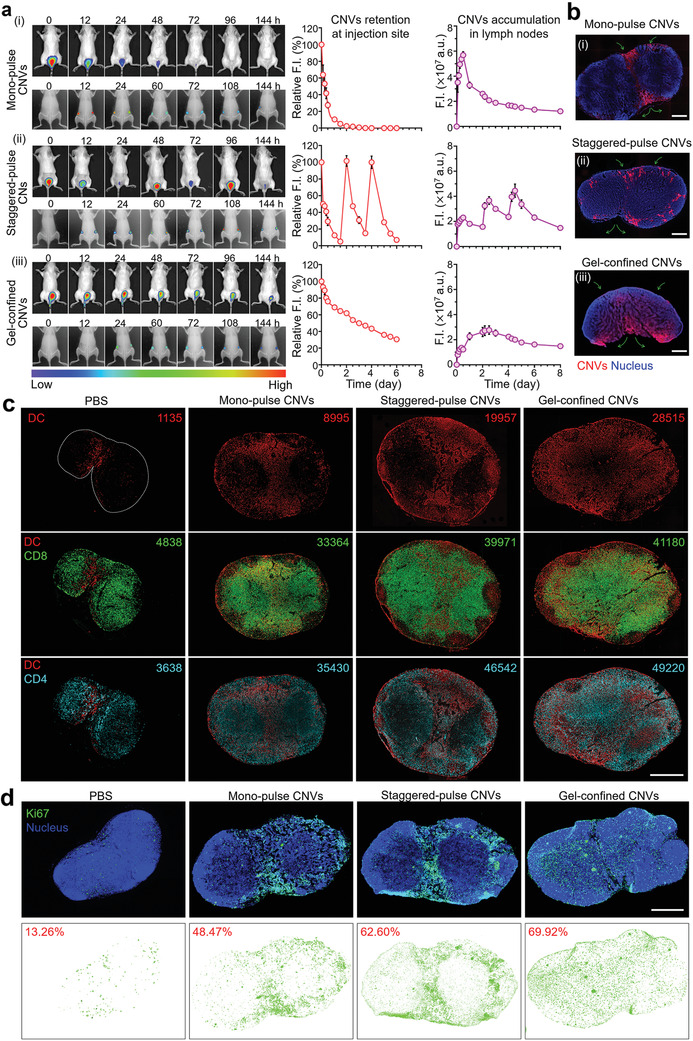
Characterizations of the lymph node delivery, intralymph node distribution, and subsequent immunoresponses elicited by three delivery modes for CNVs. a) In vivo imaging analysis for F.I. of 1,1‐dioctadecyl‐3,3,3,3‐tetramethylindotricarbocyanine iodide (DiR)‐labeled CNVs retention at injection site (top, back imaging) and accumulation of lymph nodes (bottom, abdominal imaging) in mice treated via the three delivery modes. Noted that the relative quantitative F.I. of CNVs were normalized to themselves in each group. a.u. represent arbitrary unit. b) Fluorescence images of lymph node frozen sections. The orientation of green arrows indicated the location of afferent lymph flow and efferent lymph flow of each lymph node. Scale bar: (i) 300 µm; (ii) 400 µm; (iii) 500 µm. c) Multiple immunofluorescence analysis of lymph node sections at day 7 postvaccination and corresponding quantitation of cell phenotypes in each slide (top: DCs, middle: CD8 T cells, bottom: CD4 T cells) using Inform Image Analysis software (PerkinElmer). DCs (red), CD8 T cells (green), and CD4 T cells (cyan). Scale bar: 1 mm. d) Immunofluorescence analysis of Ki67 expression. The percentages indicated the ratio of the area of proliferating cells (Ki67) to the total area of lymph nodes. Ki67 (green) and nucleus (blue). Scale bar: 1 mm. Data in (a) represent the mean ± s.e.m. (*n* = 3).

After s.c. injection into the backs of mice in the mono‐pulse CNVs group, the CNVs quickly traveled to lymph nodes without APCs recruitment (**Figure** **3**a i; Figure S7, Supporting Information), and their fluorescence signal intensity gradually increased in lymph nodes before reaching a peak at 12 h. This was accompanied by a consistent decrease in the CNVs signal intensity at the injection site. For the staggered‐pulse CNVs delivery mode, APCs recruitment was not detected either (Figure S7, Supporting Information). Instead, we observed three peaks of lymph node accumulation, each corresponding to the 12 h time point following the three injections (Figure 3a ii). Note that the kinetics were highly similar for both of these passive delivery modes. In contrast, for mice in gel‐confined CNVs group, we detected an extended retention window for the CNVs at the injection site, accompanied with a rapid and persistent APCs recruitment (Figures 2f and 3a iii; Figure S7, Supporting Information). As a result, the fluorescence signal intensity for CNVs in lymph nodes was sustained at a similarly elevated level throughout the 8 d experimental time course.

When we examined lymph node sections via fluorescence microscopy, we observed distinct CNVs localization patterns resulting from each of the three distinct delivery modes. Specifically, we found that CNVs from the mono‐pulse CNVs delivery mode predominantly localized at the area nearby the afferent and efferent lymph vessels (Figure 3b i; Figure S8a, Supporting Information). The extended time window of the staggered‐pulse CNVs delivery mode resulted in a more peripheral localization pattern, with more CNVs evident in multiple marginal regions of the subcapsular sinus, although still largely outside the paracortex (Figure 3b ii, Figure S8a, Supporting Information). For the actively delivered CNVs from the gel‐confined CNVs delivery mode, we observed rich accumulation of CNVs throughout the central paracortex of lymph nodes, with especially pronounced accumulation in the T cell zone (Figure 3b iii; Figure S8a, Supporting Information). These highly distinct distribution patterns for the CNVs strongly suggested that the different delivery modes might substantially alter the extent and localization of DC–T cell interactions in lymph nodes.

To investigate whether the different intralymph node distribution patterns of CNVs resulting from the different delivery modes could modulate the distributions of immune cells inside lymph nodes, we isolated lymph nodes, photographed them, and then prepared paraffin sections for multicolor immunofluorescence staining of DCs, CD4 T cells, and CD8 T cells. We observed that the distributions of DCs in lymph nodes matched the patterns we had observed for CNVs in lymph nodes for each of the three delivery modes (Figure 3c). Thus, although there was clear DC accumulation in mice of all three delivery modes, the increased paracortex infiltration of the gel‐confined CNVs delivery mode clearly enabled more extensive DC–T cell interactions (Figure 3c; Figure S8b, Supporting Information). And we noted that the numbers of CD8 T and CD4 T cells in three delivery modes treated lymph nodes increased compared to controls. Besides, we also observed the migration of CD4 T cells into germinal centers in both three delivery modes (Figure S8b, Supporting Information).

Above distinct T cell response promoted us to examine cell proliferation in lymph nodes with immunostaining against Ki67, a biomarker of cell proliferation.^[^ ^54^ ^]^ As shown in Figure 3d, CNVs treatments increased proliferation substantially, and did so in spatial patterns consistent with the aforementioned CNVs and DC patterns. Particularly, the region of cell proliferation was larger and more localized in the paracortex in gel‐confined CNVs group, while the region of cell proliferation major in the areas outside the paracortex (nearby the afferent and efferent lymphatic vessel) in staggered‐pulse CNVs and mono‐pulse CNVs groups. Consistently, compared to the phosphate buffer saline (PBS) control group, there were substantial increases in dimensions and measured mass of lymph nodes after all three of the CNVs delivery modes, in the following order: mono‐pulse CNVs (2.2‐fold increase in mass over the control), staggered‐pulse CNVs (3.2‐fold increase), gel‐confined CNVs (3.7‐fold increase) (Figure S9, Supporting Information). Collectively, these results supported that the localization patterns for CNVs and their attendant impacts on DC–T cell interactions indeed impacted the nature of the immune responses that could be elicited by vesicles bearing an identical antigen.

### 2.4 Primary Tumor Inhibition of CNVs Vaccination under Three Delivery Modes

**Figure 4:**
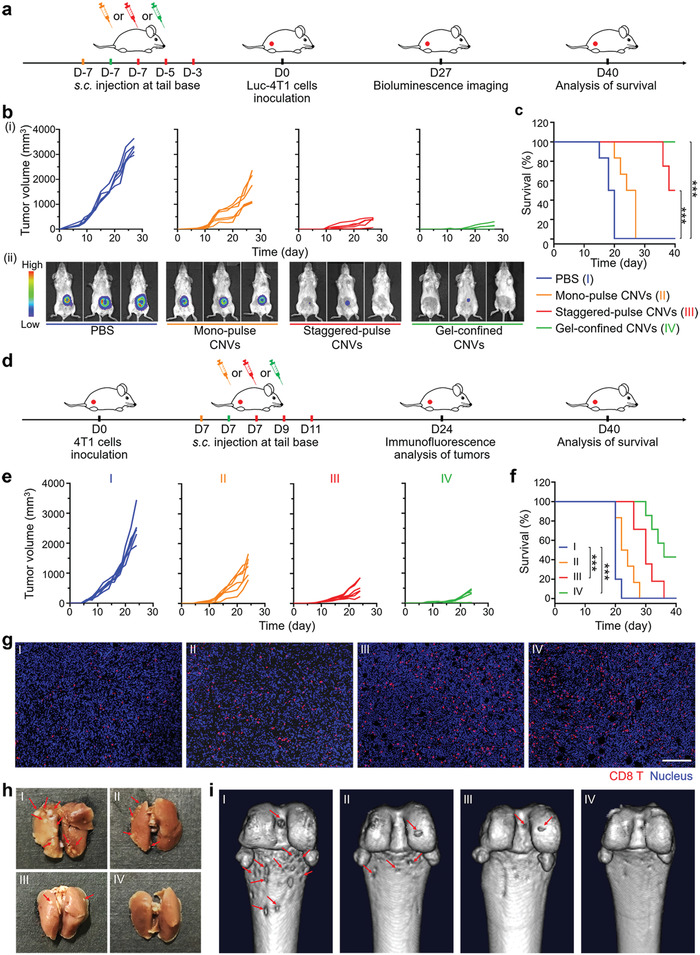
Prophylactic and therapeutic effects on 4T1 primary tumor model under vaccination with three CNVs delivery modes. a) Experimental design for a prevention of 4T1 primary tumor based on three CNVs delivery modes. Mice were prevaccinated at day ‐7 with mono‐pulse CNVs (orange) or gel‐confined CNVs (green) modes, or at days ‐7, ‐5, and ‐3 with staggered‐pulse CNVs (red), followed by s.c. injection with 5 × 10^5^ luc‐4T1 tumor cells at day 0. b) Individual tumor growth curves for mice after different treatments (i) (*n* = 6) and representative bioluminescence image analysis of tumors at day 27 (ii). c) Survival curves for mice of the different delivery mode groups (*n* = 6). d) Experimental design for a therapy of 4T1 primary tumor based on the three CNVs delivery modes. Mice were inoculated with 5 × 10^5^ 4T1 tumor cells at day 0 and subsequently vaccinated at day 7 with the mono‐pulse CNVs (orange) or gel‐confined CNVs (green) modes, or at days 7, 9, and 11 with staggered‐pulse CNVs mode (red). e) Individual tumor growth curves for mice after different treatments (*n* = 6). f) Survival curves for mice after different treatments (*n* = 6). g) Immunofluorescence image analysis of the infiltration of CD8 T cells into tumors. CD8 T cells (red) and nucleus (blue). Scale bar: 100 µm. h) Representative photographs of ex vivo lungs for analyzing pulmonary metastasis (red arrows). i) Representative images of tibia metastasis (red arrows) via computed tomography (CT). Survival analyses in (c) and (f) were calculated by log‐rank test. ***P < 0.001.

Having confirmed the spatiotemporal distribution of CNVs and distinct immune response of lymph nodes under different delivery modes, we next explored the antitumor effect of CNVs in these three delivery modes. We used the luciferase expressing 4T1 (luc‐4T1) primary tumor model. Mice were subcutaneously pretreated with monopulse CNVs, staggered‐pulse CNVs, and gel‐confined CNVs prior to 4T1 tumor inoculation and then assessed tumor size every 2 or 3 d for four weeks to assess the prophylactic tumor effects of the three nanovaccine delivery modes (**Figure** **4**a). Compared to PBS control, mice of the monopulse CNVs group showed a slight inhibition of tumor growth, while the staggered‐pulse CNVs and gel‐confined CNVs groups showed an obvious delay of tumor growth (Figure 4b i). The prophylactic efficacy was so strong that two of the six staggered‐pulse CNVs treated mice and four of the six gel‐confined CNVs treated mice had no obvious tumor burden at day 27. Moreover, bioluminescence imaging at day 27 confirmed the results from our caliper‐based analyses throughout the experimental time window (Figure 4b ii). Notably, assessment of body temperature, weight, serum cytokine levels (IL‐6, TNF‐*α*, and IFN‐*γ*), and organ histology indicated that none of the three delivery modes for the CNVs elicited abnormalities after vaccination with three CNVs delivery modes, indicating the apparent safety of CNVs vaccination protocol (Figures S10–S12, Supporting Information). Moreover, the serum biochemistry analysis revealed the good health of CNVs‐treated mice, especially for staggered‐pulse CNVs and gel‐confined CNVs groups (Figure S13, Supporting Information), and it was highly notable that the survival time of mice in these two groups was significantly prolonged until 40 d (Figure 4c).

For evaluation of therapeutic tumor efficacy, 4T1 tumor‐bearing mice (after 7 d inoculation with 4T1 cells) were given the monopulse CNVs, staggered‐pulse CNVs, and gel‐confined CNVs treatments (Figure 4d). Tumor volumes were measured every 2 or 3 d. We did observe therapeutic effects, and these followed the same trend as for the prophylactic effects. That is, the most pronounced delay in tumor progression and higher survival rates were observed for the gel‐confined CNVs group, followed by the staggered‐pulse CNVs and mono‐pulse CNVs groups (Figure 4e,f). The intensity of the therapeutic effects was mirrored in our observations for the rates of CD8 T cells infiltration in tumors (Figure 4g; Figure S14, Supporting Information). Given the frequent occurrence of metastases with the 4T1 tumor model, we were also able to assess the impacts of the three delivery modes on metastasis. The metastatic foci in the lung and serious metastasis‐induced erosion in the tibia were observed in the PBS control and mono‐pulse CNVs treated mice, while there was less erosion in the staggered‐pulse CNVs and nothing in gel‐confined CNVs treated mice (Figure 4h,i). Meanwhile, the serum biochemical analysis revealed that CNVs treatments in three delivery modes efficiently prevented organ dysfunction (Figure S15, Supporting Information).

### 2.5 Antitumor Recurrence and Metastatic Performance of CNVs under Three Delivery Modes

**Figure 5:**
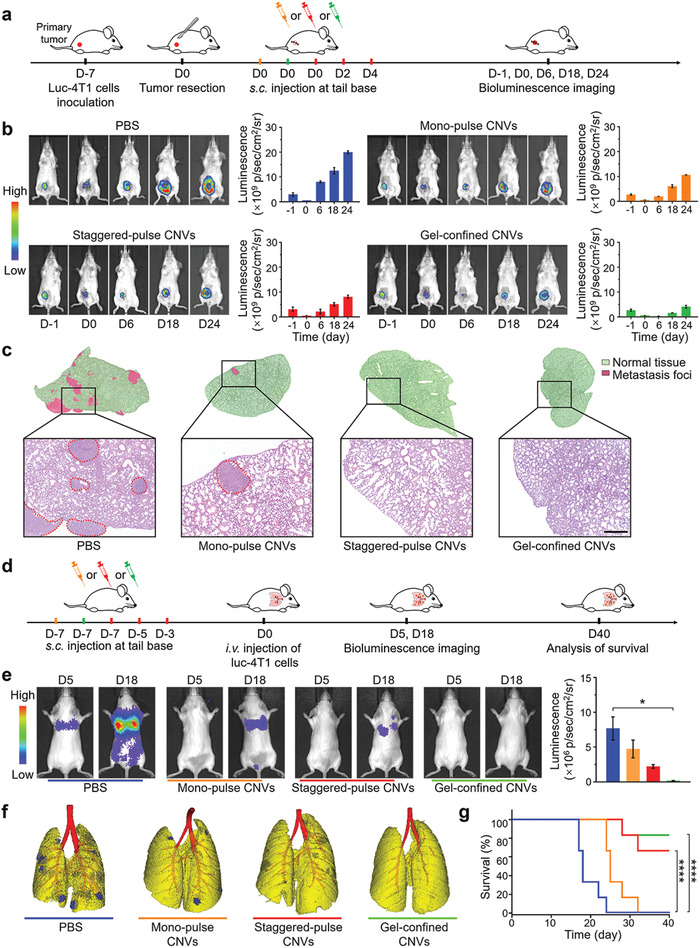
Antire‐currence performance and anti‐metastatic effects of the three CNVs delivery modes. a) Experimental design for a tumor therapy in an incomplete‐surgery luc‐4T1 tumor model. 4T1 tumor‐bearing mice received surgical tumor resection at day 0, and were subsequently vaccinated at day 0 with mono‐pulse CNVs (orange) or gel‐confined CNVs (green), or at days 0, 2, and 4 with staggered‐pulse CNVs (red) (as a treatment to inhibit postresection tumor recurrence). b) Representative bioluminescence images (left) and fluorescence quantification (right) of the primary tumor before tumor resection and recurrent tumors after resection after different treatments. c) Representative H&E stained lung sections from mice of the different treatment groups. The red circles indicated the regions of metastasis foci. Scale bar: 100 µm. d) Experimental design for an anti‐metastatic tumor therapy based on three CNVs delivery modes. Mice were pretreated at day ‐7 with monopulse CNVs or gel‐confined CNVs, or at days ‐7, ‐5, and ‐3 with staggered‐pulse CNVs, and followed by i.v. injection with 5 × 10^5^ luc‐4T1 cells at day 0. e) Representative bioluminescence images of hematogenous metastasis in lungs with different CNVs prevaccination delivery modes and corresponding quantitative luminescence signals of lung metastasis. f) In vivo imaging analysis of lung metastasis via CT. Yellow indicated normal tissue while blue indicated metastasis foci. g) Survival curves for mice of the different delivery mode groups (*n* = 6). Data represent the mean ± s.e.m. (g, *n* = 6; e, *n* = 3). *P*‐values were calculated via one‐way ANOVA with Tukey post‐hoc test. *P < 0.05. Survival analysis in (g) was calculated by log‐rank test. ****P < 0.0001.

Looking beyond study of primary tumors, we next evaluated the antitumor recurrence performance of CNVs as administered via the different delivery modes using a tumor recurrence model. Mice were subcutaneously injected with luc‐4T1 cells, and the majority of the tumor materials were surgically resected on post‐tumor cell inoculation day 7, after which mice received the CNVs via the three delivery modes (**Figure** **5**a). Bioluminescence imaging revealed reduced tumor recurrence for all three delivery modes compared to the PBS control, with the extent of inhibition following the same trend that we observed for the prophylactic and the therapeutic models (Figure 5b). Specifically, the significantly delayed recurrent tumor growth was observed in the gel‐confined CNVs group, exhibiting 78% tumor growth inhibition rate (TGI) while 60% TGI and 44% TGI for staggered‐pulse CNVs and mono‐pulse CNVs groups, respectively. We also assessed lung metastasis in this model and found that there were almost no metastatic nodules in H&E‐stained lung sections prepared from gel‐confined CNVs or staggered‐pulse CNVs treated mice (Figure 5c), suggesting that these nanovaccine delivery modes also succeeded in preventing lung metastasis.

To further analyze the anti‐metastatic effects of the CNVs as administered via the different delivery modes, we established a classic hematogenous metastatic model with luc‐4T1 cells (Figure 5d).^[^ ^55^ ^]^ Specifically, prior to the intravenous (i.v.) infusion of luc‐4T1 cells at day 0, mice were given prevaccination via the three delivery modes (from day ‐7). Bioluminescence imaging on metastasis model at days 5 and 18 revealed that PBS control showed obvious lung metastasis at day 5; no metastasis signal was detected for any of the three CNVs groups at day 5 (Figure 5e). At day 18, the PBS control mice had extremely strong signals for lung metastasis, as well as other metastases throughout the body (Figure 5e). Analysis of the various CNVs group mice at day 18 revealed a complete absence of lung metastasis in the gel‐confined CNVstreated mice, while the other two showed metastases to differing extents: mice of the mono‐pulse CNVs group had developed substantial lung metastasis and the staggered‐pulse CNVs treated mice had some metastatic nodules forming (Figure 5e). In vivo imaging analysis of the same mice via CT on day 18 further confirmed these trends, again showing that the gel‐confined CNVstreated mice had no detectable metastatic nodules at this point (Figure 5f). We also tracked the survival of the metastasis model mice, and found that the mice of the three CNVs groups survived longer than the control mice, with an especially pronounced increase for the gel‐confined CNVs treated mice, 83.3% of which were still alive at the defined endpoint of 40 d (Figure 5g).

### 2.6 Extension of Testbed into Liposome‐Based Nanovaccines for Comparative Evaluation of Three Delivery Modes

**Figure 6:**
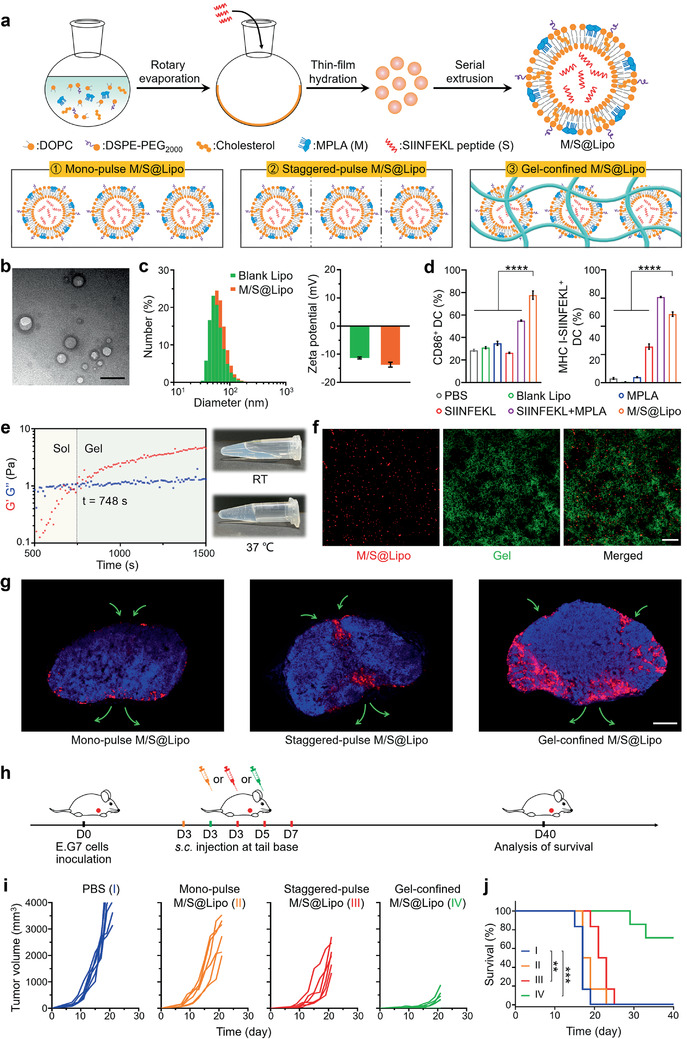
Synthesis, characterizations, intralymph node distribution, and antitumor effect of M/S@Lipo under three delivery modes. a) Schematic illustration of the construction of M/S@Lipo (top) and their three delivery modes (bottom). b) TEM image of M/S@Lipo. Scale bar: 100 nm. c) Size distributions and zeta potentials of blank liposomes (blank Lipo) and M/S@Lipo. d) The expression of costimulatory molecules CD86 and antigen presentation (MHC I‐SIINFEKL) on BMDCs after 24 h incubation with PBS, blank Lipo, MPLA, SIINFEKL peptide, SIINFEKL peptide plus MPLA, or M/S@Lipo. e) The evolution of *G*′ and *G*″ of gel‐confined M/S@Lipo at 37 °C (left) and photographs of the gel‐confined M/S@Lipo before and after incubation at 37 °C (right). f) CLSM images of gel sample for gel‐confined M/S@Lipo in vitro. M/S@Lipo (red) and hydrogel (green). Scale bar: 50 µm. g) Fluorescence images of lymph nodes frozen section at day 7 after three M/S@Lipo delivery modes treatments. Scale bar: 500 µm. h) Experimental design for a therapy of E.G7 primary tumor based on the three M/S@Lipo delivery modes. Mice were inoculated with 5 × 10^5^ E.G7 cells at day 0 and subsequently vaccinated at day 3 with the mono‐pulse M/S@Lipo (orange) or the gel‐confined M/S@Lipo (green), or at days 3, 5, and 7 with the staggered‐pulse M/S@Lipo (red). i) Individual tumor growth curves for E.G7 tumor‐bearing mice after different treatments (*n* = 6). j) Survival curves for mice of the different delivery mode groups (*n* = 6). Data in (c) and (d) represent the mean ± s.e.m. (*n* = 3). *P*‐values were calculated via one‐way ANOVA with Tukey post‐hoc test. ****P < 0.0001. Survival analysis in (j) was calculated by log‐rank test. **P < 0.01, ***P < 0.001.

Beyond tumor‐derived vesicle‐based nanovaccines, we wonder the performance of other nanovaccine materials in three delivery modes. Here, we chose liposomes to serve as another nanovaccine testbed. Liposome‐based nanovaccines composed of lipid content including 1, 2‐dioleoyl‐sn‐glycero‐3‐phosphocholine (DOPC), 1, 2‐distearoyl‐sn‐glycero‐3‐phosphoethanolamine‐[amino(polyethyleneglycol)_2000_] (DSPE‐PEG_2000_), cholesterol, immunostimulator monophosphoryl lipid A (MPLA), and ovalbumin antigenic peptide (SIINFEKL) were prepared through lipid film hydration and membrane extrusion method (0.2 and 0.05 µm), termed as M/S@Lipo (**Figure** **6**a). TEM and DLS analysis together showed that the M/S@Lipo had an average diameter of 63.4 nm and negative charge of 13.7 mV, all of which had no significant changes compared to blank liposomes (Figure 6b,c). Although the size distribution of M/S@Lipo was a little larger than CNVs (Figures 1b and 6c), their diameters (<200 nm) still ensure their lymph node targeting ability.^[^ ^56^, ^57^ ^]^ Moreover, a tiny size increase (<10 nm) of liposome as compared to CNVs should not have big impact on their lymph node accumulation and intralymph node distribution. The final content of MPLA in the liposome was 14.30% (wt% of liposomes) and the encapsulation rate of the SIINFEKL peptide was 74.49%. There were almost no notable changes of size and zeta potential for M/S@Lipo storage at 4 °C in PBS for two weeks, suggesting the good stability of M/S@Lipo (Figure S16, Supporting Information).

Having successfully constructed M/S@Lipo, we next investigated whether they had the immune activation ability for DCs in vitro. Indeed, the level of the costimulatory marker CD86 and the antigen presentation (MHC I‐SIINFEKL) were significantly increased for the DCs incubated with M/S@Lipo (Figure 6d). Collectively, these results suggested that M/S@Lipo nanovaccines with appropriate size and sufficient DCs activation ability were suitable for using as another testbed in comparing three delivery modes. Thus, we continued to test the feasibility of gel‐confined system for embedding M/S@Lipo. When we mixed the M/S@Lipo with hydrogel in 3:7 volume ratio at RT, a gel formation of the mixture was observed within 748 s at 37 °C, with M/S@Lipo embedded in its porous cavities (Figure 6e,f). These results demonstrated the successful development of gel‐confined M/S@Lipo, which could once again be used as the APCs‐mediated active delivery mode in further comparison of three delivery modes.

Given the establishment of M/S@Lipo nanovaccine testbed and gel‐confined M/S@Lipo delivery mode, we next investigated intralymph node distribution of M/S@Lipo in three delivery modes. Briefly, 1,1‐dioctadecyl‐3,3,3,3‐tetramethylindodicarbocyanine perchlorate (DiD)‐labeled M/S@Lipo were subcutaneously injected into the back of mice in three delivery modes, and the lymph nodes were isolated for fluorescent observation. Intriguingly, we found that the M/S@Lipo localization patterns were also different resulted from the three delivery modes (Figure 6g). Specifically, M/S@Lipo were mostly localized at the regions of the subcapsular sinus for monopulse M/S@Lipo delivery mode, while M/S@Lipo also localized nearby afferent and efferent lymphatic vessels and at the regions of the subcapsular sinus for staggered‐pulse M/S@Lipo delivery mode. For gel‐confined M/S@Lipo delivery mode, we observed more M/S@Lipo infiltrated into the central paracortex of lymph nodes (Figure 6g). The result illustrated the versatility of the three delivery modes whether for M/S@Lipo or CNVs.

After verifying the similar trends of distribution of M/S@Lipo and CNVs in three delivery modes, we next established E.G7 tumor model to evaluate the therapeutic effects of M/S@Lipo in three delivery modes. Mice were subcutaneously inoculated with E.G7 cells at day 0 and subsequently given mono‐pulse M/S@Lipo (day 3), staggered‐pulse M/S@Lipo (days 3, 5, and 7), and gel‐confined M/S@Lipo (day 3) treatments (Figure 6h). Tumor volumes were measured every 2 or 3 d. Compared to PBS control, the most pronounced tumor inhibition was observed for mice in the gel‐confined M/S@Lipo group (TGI = 90% at day 21), followed by the staggered‐pulse M/S@Lipo (TGI = 54%) and mono‐pulse M/S@Lipo groups (TGI = 37%) (Figure 6i). Further, it was notable that the survival time was significantly improved in gel‐confined M/S@Lipo treated mice (66.7% of mice were still alive at day 40) (Figure 6j). These results again demonstrated the importance of choosing a suitable delivery mode for superior antitumor immunotherapy.

## 3 Conclusion

This study rigorously demonstrated the profound influence that delivery mode had on the ultimate immunomodulatory effects of a nanovaccine. Our work with tumor‐derived vesicle based nanovaccines in a variety of tumor models illustrated how nanovaccine delivery modes (e.g., passive vs active) altered the intralymph node distribution of nanovaccines and thereby altered DC–T cell interactions for inducing distinct immunoresponse for immunotherapy efficacy. Specifically, CNVs distribution nearby the afferent and efferent lymph vessels in mono‐pulse CNVs delivery mode only induced a weak immuno proliferation at this area, leading to a slightly inhibition of tumor growth. In terms of staggered‐pulse mode, CNVs were evident in multiple marginal regions of the subcapsular sinus owing to the extended time window, causing extended region of cell proliferation compared with monopulse mode and achieving moderate tumor inhibition. Once the CNVs were formulated with gel, this gel‐confined mode exhibited a rich accumulation of CNVs throughout the paracortex, leading to the region of cell proliferation throughout this area and resulting in the significant delay of tumor growth. Besides, we also tested the versatility of other nanovaccine materials in three delivery modes using liposome‐based nanovaccines, and similar trend of intralymph nodes distribution was observed for liposomes. It was important to emphasize that the focus of our study was on delivery mode per se, rather than identifying an optimal one‐size‐fits‐all delivery mode for the nanovaccinology field. Meanwhile, we anticipated that a non‐gel‐based delivery mode could have outperformed the hydrogel‐confined mode we used if the nanomaterial under study was further designed with conjugation with particular modified molecule, or with some other immune reactivity or property.^[^ ^58^ ^]^ At minimum, it was clear that selection of delivery mode should become an integral step in the experimental designs of nanomedicine studies.

The very large differences we observed with the delivery modes we tested raised an interesting question about previously reported performance for nanovaccines. In light of our data, it seemed probable that the reported performance for many nanomaterials was actually suboptimal, and could be substantially increased the optimization of delivery. This point was especially salient regarding nanovaccines derived from patient tumors. Given the extreme rarity of such materials for generating personalized nanovaccines, careful optimization of delivery modes could substantially improve resource utilization and potentially patient outcomes.

Although we herein have successfully demonstrated profound impacts of nanovaccine delivery mode on the intralymph node spatiotemporal distribution and immunotherapy efficacy, efforts can be made for further optimization of immune response in the future. For example, more nanovaccine's parameters (such as the injection dose/interval/frequency) and gel properties (such as rheology/porosity/adjuvanticity) can be tailored to enable yet‐further fine‐tuning of immuno‐dynamics. Furthermore, considering the better understanding about the particular influences of a given delivery mode on a nanovaccine's immunomodulatory performance, we should in theory be able to tailor combinations of delivery modes to enhance vaccination performance. In addition, given the important role of immune checkpoints for tumor immune resistance, combinational use of their inhibitors (such as programed death 1 antibody) also deserves investigation, and the extent of therapeutic benefits may vary among the delivery modes.

## 4 Experimental Section

##### Materials and Reagents

The sequence of CpG is 5′‐TCCATGACGTTCCTGACGTT‐3′. CpG, CpG capped with an aldehyde end group (CpG‐CHO) and fluorescein‐conjugate CpG‐CHO were synthesized by Sangon Biotech Co., Ltd. (Shanghai, China). All primary antibodies and secondary antibodies were purchased from Abcam (Cambridge, UK). CB were purchased from Aladdin (Shanghai, China). Fetal bovine serum (FBS), penicillin‐streptomycin, and all medium for cell culture were purchased from Gibco (Carlsbad,USA). Recombinant mouse GM‐CSF and IL‐4 were purchased from Peprotech (Rocky Hill,USA). All fluorochrome‐conjugated antimouse antibodies and ELISA kits were purchased from eBioscience (San Diego, USA). Cytometric bead array (CBA) mouse inflammation kit was purchased from BD Biosciences (San Jose,USA). Fluorescein isothiocyanate (FITC), LysoTracker Red DND‐99, Live/Dead viability kit, Hochest 33258, optimal cutting temperature (OCT) compound, tissue protein extraction reagent (T‐Per) and Halt protease inhibitor cocktail, Micro BCA protein assay kit, and Luminex multiplex assay kit were purchased from Thermo Fisher Scientific (Waltham, USA). Fluorescent dyes (DiD, DiR, and Cy7‐SE) and d‐luciferin (potassium salt) were obtained from Fanbo Biochemicals Co., Ltd. (Beijing, China). CS was purchased from Xi'an TianBao Biological Technology Co., Ltd. (Xi'an, China). CH was synthesized in the lab using CS by a modified method.^[^ ^59^ ^]^ CG was purchased from NovaMatrix (Sandvika, Norway). *α*, *β*‐Glycerophosphate was provided by Kaiyuan Pharmaceutical & Chemical Co., Ltd. (Xi'an, China). DOPC and MPLA were purchased from Sigma‐Aldrich (St. Louis,USA). DSPE‐PEG_2000_ and cholesterol were purchased from Xi'an Ruixi Biological Technology Co., Ltd. (Xi'an, China). SIINFEKL peptides were synthesized by Ji Er Biochemical Co., Ltd. (Shanghai, China). Coomassie Brilliant Blue dye was purchased from Solarbio Science & Technology Co., Ltd. (Shanghai, China). Cell membrane protein extraction kit was purchased from Beyotime Biotechnology Co., Ltd. (Beijing, China). Lysosome Isolation Kit was purchased from BestBio Technology Co., Ltd. (Shanghai, China). All other reagents were of analytical grade.

##### Cell Lines and Animal Models

4T1, luc‐4T1 breast carcinoma, and E.G7 murine lymphoma cell lines were obtained from the American Type Culture Collection. 4T1 and luc‐4T1 cells were cultured in RPMI‐1640 medium supplemented with FBS (10%, v/v) and penicillin‐streptomycin (1%, v/v) at 37 °C in a humidified atmosphere containing 5% CO_2_. E.G7 were cultured in high glucose RPMI‐1640 medium supplemented with FBS (10%, v/v), penicillin‐streptomycin (1%, v/v), 2‐mercaptoethanol (0.05 × 10^−3^ м), and G418 (0.4 mg mL^−1^) at 37 °C in a humidified atmosphere containing 5% CO_2_. All mice used in this study were female Balb/c mice (6–8 weeks) and male C57BL/6 mice (6–8 weeks) from Vital River Laboratories (Beijing, China). All animal works were approved by the Institutional Animal Care and Use Committees at the Institute of Process Engineering (Approval ID: IPEAECA2016158). The animal experiments were conducted under the guidelines of the Regulations for the Care and the Use of Laboratory Animals (China, GB/T 35892‐2018).

##### Preparation of CNVs and M/S@Lipo Nanovaccines—*CNVs nanovaccines*

To produce MVs, 4T1 cells were first cultured in a 75 cm^2^ petri dish until 90% confluence, and then recultured with fresh serum‐free RMPI‐1640 medium (2 mL) containing CB (10 µg mL^−1^) at 37 °C for 30 min.^[^ ^34^ ^]^ Cells and formed MVs were detached through trypsin digestion. The resultant cell/MVs suspensions underwent vigorous vortex for 1 min and then mixed with same volume of FBS. Afterward, the suspensions were centrifuged at 200 *g* for 5 min to remove cells, and the resultant supernatant was further centrifuged at 2000 *g* for 20 min to harvest MVs. MVs were resuspended in PBS solution and extruded through polycarbonate membrane (0.08, 0.2, and 0.05 µm) to form NVs. Various amount of CpG‐CHO was incubated with 1 mg NVs solution at 37 °C for 4 h to obtain CNVs. The unconjugated CpG‐CHO were removed through ultrafiltration and then followed by PBS wash at least five times.

*Preparation of CNVs and M/S@Lipo Nanovaccines—M/S@Lipo nanovaccines*: Typical film dispersion method was used to synthesize M/S@Lipo.^[^ ^60^ ^]^ 1 mg DOPC, 0.33 mg DSPE‐PEG_2000_, 0.33 mg cholesterol, and 0.05 mg MPLA were mixed in chloroform/methanol (3:1, v/v, 20 mL) solution. Afterward, the mixture was evaporated to a thin film on a rotary evaporator. The as‐prepared thin film was further dried under vacuum overnight to completely remove solvent and rehydrated in the PBS solution (1 mL) containing SIINFEKL peptides (0.5 mg mL^−1^) under sonication at 37 °C. Ultimately, the mixture was extruded through polycarbonate membrane (0.2 and 0.05 µm) at 55 °C to form uniform M/S@Lipo. For fluorescent M/S@Lipo preparation, fluorescent dye DiD was added into the chloroform/methanol mixture before evaporation process. The excess contents and dye were removed through aforementioned ultrafiltration procedure. Similarly, blank Lipo was prepared with DOPC, DSPE‐PEG_2000_, and cholesterol following the abovementioned process. The contents of MPLA and SIINFEKL peptide were detected using reverse phase‐high performance liquid chromatography (RP‐HPLC).

All procedures for preparation of CNVs and M/S@Lipo nanovaccines were conducted under sterile conditions.

##### Characterizations of CNVs and M/S@Lipo Nanovaccines

The fluorescent 4T1 cells were obtained after incubation with DiD (10 µg mL^−1^) in PBS solution (2 mL) and then cells were treated with CB (10 µg mL^−1^) according to procedure described previously to prepare fluorescent MVs. Afterward, fluorescent NVs were obtained via extrusion of these fluorescent MVs. CB‐treated fluorescent 4T1 cells, MVs, and NVs were observed by CLSM (SP50, Leica). To prepare fluorescent CNVs, 1 mg DiD‐labeled NVs were incubated with various amounts of fluorescein‐conjugated CpG‐CHO at 37 °C for 4 h. The fluorescent CNVs were imaged by SIM with DeltaVision OMX V3. The morphology of CNVs and M/S@Lipo was characterized by TEM using JEM‐2100F and JEM‐1400F, respectively. Their sizes and zeta potentials were measured by DLS using ZetaSizer Nano Series (Malvern).

##### SDS‐PAGE and Western Blotting

The membrane proteins of tumor cells, MVs, and CNVs were prepared by using cell membrane protein extraction kit and further determined by the BCA assay kit. Afterward, all samples with equal protein amount were added into the wells and separated in 10% acrylamide/bisacrylamide gel. For SDS‐PAGE analysis, the gel was stained with Coomassie Brilliant Blue dye and imaged by MF‐ChemiBIS 3.2 imaging system (DNR). For Western blotting analysis, proteins in the gel were transferred to a polyvinylidene fluoride membrane, which were then incubated with primary antibodies (Na^+^/K^+^ ATPase‐*α*1, CD44 ,CD47, or EPCAM) (1:1000) at 4 °C overnight, followed by incubation with horseradish peroxidase (HRP)‐conjugated secondary antibodies (1:5000) at RT for 2 h. Proteins were visualized with HRP‐enhanced chemiluminescence (ECL) and imaged by MF‐ChemiBIS 3.2 imaging system (DNR).

##### DCs Uptake and Activation In Vitro

BMDCs were derived from bone marrow cells using standard protocol.^[^ ^61^ ^]^ Briefly, bone marrow cells were isolated from the femurs and tibias of mice, and then cultured in RPMI‐1640 medium containing FBS (10%, v/v), penicillin‐streptomycin (1%, v/v), GM‐CSF (10 ng mL^−1^), and IL‐4 (20 ng mL^−1^) at 37 °C in a humidified atmosphere containing 5% CO_2_. After 5 d, BMDCs were collected for further use.

To investigate DCs uptake, MVs, NVs, and CNVs were prelabeled with fluorescent dye DiD (10 µg mL^−1^). The total protein amounts of MVs, NVs, and CNVs were quantified by using Micro BCA protein assay kit. After 6 h incubation with 10 µg protein amounts of MVs, NVs, NVs plus CpG (2 µg), or CNVs (containing 2 µg CpG), BMDCs were collected and stained with FITC‐CD11c antibody for flow cytometry analysis.

To evaluate DCs activation ability of CNVs nanovaccines, BMDCs were incubated with nonlabeled formulations aforementioned for 24 h. Then cells were collected and stained with FITC‐CD11c, PE‐CD40, APC/Cy7‐CD86, eFluor 450‐MHC II, BV510‐MHC I, and APC‐CD80 antibodies. Meanwhile, the corresponding cell culture supernatants of BMDCs were collected to detect the secretion levels of IL‐6, IL‐12, TNF‐*α*, and MCP‐1 using CBA mouse inflammation kit.

Similarly, BMDCs incubated with blank Lipo, MPLA (1 µg), SIINFEKL peptide (10 µg), SIINFEKL peptide plus MPLA, or M/S@Lipo (containing 1 µg MPLA and 10 µg SIINFEKL peptide) for 24 h were collected and stained with FTIC‐CD11c, APC‐MHC I‐SIINFEKL, and APC/Cy7‐CD86 antibodies to detect the expression of CD86 and MHC I‐SIINFEKL.

All stained samples in above experiments were detected by CytoFLEX LX flow cytometer (Beckman Coulter), and the data were analyzed by CytExpert software.

##### Evaluation of CNVs Release Behavior

Lysosomes were isolated from BMDCs according to the manufacturer's instructions of Lysosome Isolation Kit. Afterward, the resulting lysosomes were processed with freeze–thaw to prepare lysosomal inclusion for evaluation of CpG release in vitro. Briefly, fluorescein‐CpG‐modified NVs were incubated in lysosomal inclusion at 37 °C, and then the released fluorescein‐CpG was collected via ultrafiltration and further detected at different time points using enzyme microplate reader (TECAN, Infinite F2000). To invesigate the intracellular fate of CpG and NVs, BMDCs were stained with LysoTracker Red DND‐99 after 12 h incubation with fluorescein‐CpG‐modified, DiD‐labeled NVs. Afterward, fluorescence observation of DC sample was done by CLSM (SP50, Leica).

##### Preparation of Different Hydrogel and Evaluation of Their Biocompatibility and DC Recruitment Behaviors In Vivo

The gel formulations of CG, chitosan (CS), and chitosan hyamine (CH) hydrogel were prepared according to the crosslinking method as described in a previous work.^[^ ^59^ ^]^ Briefly, 1.2% (wt/v) CG was dissolved in 8 mL sterilized water at RT, and then crosslinked with *α*, *β*‐glycerophosphate (10%, wt/v, 2 mL) under stirring in an ice bath to obtain CG hydrogel. For CS and CH hydrogel preparation, 1.2% (wt/v) CS or CH was dissolved by 5 mL acetic acid (0.15 m, 5 mL), and then CS solutions were crosslinked with *α*, *β*‐glycerophosphate (15%, wt/v, 2 mL) while CH solution was crosslinked with *α*, *β*‐glycerophosphate (10%, wt/v, 2 mL) according to abovementioned process.

To assess the biocompatibility of above three gels, CS, CG, or CH gels formulations were injected on the back of mice. Afterward, gel samples were retrieved at day 3 and gently homogenized on a 40 µm cell strainer to obtain single‐cell suspensions. Then cells were stained with Live/Dead viability kit under manufacturer's instructions and then observed by CLSM.

For evaluation of DC recruitment and activation, mice were injected with CG and CS loading with/without CNVs. At day 3 postinjection, gel samples were retreived for cell isolation according to the procedure above. Afterward, the isolated cells were stained with FTIC‐CD11c, APC‐CD80, and eFlour450‐MHC II antibodies and the recruited DC (gating on CD11c^+^) and their expressions of CD80 and MHC II were determined by flow cytometry.

##### Characterizations of CG Hydrogel‐Confined Depot Delivery System

Fluorescent CG hydrogel was obtained after incubation with FITC at 4 °C overnight. The morphology and structure of chitosan hydrogel were characterized by scanning electron microscopy (SEM) using JSM‐6700F (JEOL) and CLSM at gel form. To obtain the gel‐confined depot delivery system, 660 µg protein amount of CNVs (or containing 200 µg SIINFEKL peptide of M/S@Lipo) were incorporated with liquid form of hydrogel in a volume ratio of 3:7. The encapsulation of DiD‐labeled CNVs (or M/S@Lipo) in FITC‐labeled hydrogel was further visualized by CLSM at gel form. The gelation time of blank gel (without nanovaccines) and gel‐confined CNVs (or M/S@Lipo) were determined by Rheometer (Anton‐Paar MCR302).

##### In Vivo Degradation of Gel‐Confined CNVs

CG hydrogel was labeled with Cy7 after incubation with fluorescent dye Cy7‐SE at 4 °C overnight. Mice were subcutaneously injected with the 150 µL Cy7‐labeled gel‐confined CNVs (660 µg) solution and then imaged by IVIS Spectrum Imaging System (PerkinElmer) at different time points. Quantification of Cy7 fluorescence signals was conducted with IVIS Living Image software. Some of mice were sacrificed and the skin tissues at the injection site were collected to observe the gel degradation and skin damages.

##### In Vivo Recruitment Behavior of Gel‐Confined CNVs

To visualize the cell recruitment of gel‐confined CNVs, mice were subcutaneously injected with 150 µL mixture solution of FITC‐labeled hydrogel (105 µL) and DiD‐labeled CNVs (660 µg, 45 µL), and gel sample was retrieved after 3 d. The gel sample was embedded in OCT and then was made into 10 µm frozen section using a cryostat microtome (CM1950, Leica), followed by counterstain with Hochest 33258 (5 µg mL^−1^).

For histological analysis, gel samples were retrieved from mice after 3 d injection with 150 µL gel‐confined CNVs solution or 105 µL blank CG gel solution (equal amount of chitosan). The retrieved gel samples were fixed in 4% (wt/v) formalin and then were made into 4 µm paraffin sections for H&E staining. All tissue slices above were imaged by using Vectra fluorescent scanning system and processed with Inform Image Analysis software. To quantify the APCs recruitment, cells were isolated from the retrieved gel samplesaccording to the procedures aforementioned, and then counted with a handheld automate cell counter. Afterward, cells were then stained with FTIC‐CD11c, PE‐F4/80, PE/Cy7‐CD11b, APC‐CD80, and eFlour450‐MHC II antibodies and the cell types (DCs: CD11c^+^, macrophage: CD11b^+^ F4/80^+^) and their expressions of CD80 and MHC II were determined by flow cytometry. Alternatively, to evaluate the released cytokines profiles, the cells obtained from a parallel mice experiment were digested using 350 µL T‐Per reagent under sonication, and the suspension was then centrifuged at 10 000 *g* to obtain protein supernatants. Protease inhibitor (1×) was used for protecting protein from degradation during digestion. The chemokines (IP‐10, MIP‐2, MIP‐1*α*, MIP‐1*β*, GRO‐*α*, and RANTES) and inflammatory cytokines (IL‐6, IL‐1*β*, IL‐12, TNF‐*α*, IFN‐*γ*, and GM‐CSF) levels in the supernatants were measured by using Luminex multiplex cytokine analysis kits.

To assess the viability of recruited cells, cells were isolated from the gel samples retrieved at days 3, 7, and 14 according to the procedures above. Cells were stained with Live/Dead viability kit under manufacturer's instructions and then observe by CLSM.

##### Intralymph Node Distribution of Nanovaccines and Immune Cells after Vaccination with Three Delivery Modes

The experimental design of three nanovaccine delivery modes was described as followed: Balb/c mice were subcutaneously single‐injected on the back at day 0 with mono‐pulse CNVs or gel‐confined CNVs, or multiple‐injected (days 0, 2, and 4) with staggered‐pulse CNVs. The total proteins amount of delivered CNVs was 660 µg for mice in each delivery modes. For M/S@Lipo nanovaccines, similar vaccination process was conducted with C57BL/6 mice. The total delivered amount of SIINFEKL peptides in M/S@Lipo was 200 µg for mice in each delivery.

To evaluate nanovaccine distribution in lymph nodes, CNVs (or M/S@Lipo) were labeled with fluorescent dye DiR (or DiD). For in vivo imaging, mice vaccinated with DiR‐labeled CNVs in three delivery modes were scanned using Kodak In vivo Imaging System FX Pro at different time points. The DiR fluorescence signal retention at the injection site and distribution in lymph nodes were quantified by Carestream MI SE software. For ex vivo immunofluorescence analysis, mice vaccinated with DiD‐labeled CNVs (or M/S@Lipo) in three delivery modes were sacrificed at day 7, and the inguinal lymph nodes were collected and then were made into 8 µm frozen sections. The tissue slices were counterstained with Hochest 32258 (5 µg mL^−1^) and mounted for further imaging by using Vectra fluorescent scanning system (PerkinElmer).

To assess the distribution of immune cells in lymph nodes, inguinal lymph nodes were collected from mice after 7 d vaccination with three CNVs delivery modes, follow to be photographed and weighed. For multiplex immunofluorescence staining, lymph nodes were fixed in 4% (wt/v) formalin and then were made into 4 µm paraffin sections for multiplex immunofluorescence staining. Opal multiplex IHC system was carried out according to the manufacturer's instructions. Briefly, lymph node slices were dried at 65 °C overnight followed by dewaxing with xylene, gradient dehydration with different concentrations of ethanol (100%, 95%, and 70%), microwave‐mediated antigen retrieval using the ethylene diamine tetraacetate acid (EDTA), or citrate buffer and blocking with 1% (wt/v) bovine serum albumin (BSA). The primary antibodies and corresponding detection dye were listed as next: CD11c (1:50) detected with OPAL620 (1:100), CD8a (1:200) detected with OPAL520 (1:100), and CD4 (1:400) detected with OPAL570 (1:100). Finally, counterstained and mounted were done as described before for further imaging and analysis. Slices were imaged using Vectra Polaris Automated Quantitative Pathology Imaging System (PerkinElmer) for further analyzing the distribution of specific cell phenotypes (CD11c‐DCs, CD4‐CD4 T cells, and CD8a‐CD8 T cells).

For evaluation of proliferating cells in lymph nodes, inguinal lymph nodes were made 8 µm frozen sections, followed by fixation with 4% (wt/v) formalin and blocking with 1% (wt/v) BSA. Afterward, these sections were firstly incubated with Ki67 primary antibody (1:100) at 4 °C overnight, followed by PBS wash for three times, incubation with Alex Fluor 488‐conjugate secondary antibody (1:200) at RT for 2 h. Finally, counterstained and mounted were done as described before, and slices were imaged by using Vectra fluorescent scanning system (PerkinElmer).

All images obtained in above experiments were processed and analyzed with Inform Image Analysis software.

##### 4T1 Primary Tumor Models and Treatment

To study the prophylactic effect of three CNVs delivery modes, Balb/c mice were prevaccinated with mono‐pulse CNVs (day ‐7), staggered‐pulse CNVs (days ‐7, ‐5, and ‐3), or gel‐confined CNVs (day ‐7). The total proteins amount of delivered CNVs was 660 µg in each delivery modes. Mice administrated with PBS were used for control. At day 0, luc‐4T1 cells (5 × 10^5^) were inoculated into the mammary fat pad of mice. Tumors were monitored by using digital caliper every 2–3 d and the tumor volume (*V*) was calculated via typical formula

(1) $$V=\frac{L}{}$$

where *L* is the longest and *W* is the shortest tumor diameter. At day 27, tumor burdens were observed using in vivo bioluminescence imaging system. Specifically, mice were intraperitoneal injected with 100 µL Dulbecco's phosphate buffer saline (DPBS) containing d‐luciferin (15 mg mL^−1^) and then imaged by using the IVIS Spectrum Imaging System (PerkinElmer) after 10 min.

To evaluate the therapeutic effects of three CNVs delivery modes, Balb/c mice were inoculated with 4T1 cells (5 × 10^5^) at mammary fat pad at day 0 then subcutaneously vaccinated with mono‐pulse CNVs (day 7), staggered‐pulse CNVs (days 7, 9, and 11), or gel‐confined CNVs (day 7). Tumor volume was recorded every 2–3 d as described before. The total proteins amount of delivered CNVs was 660 µg in each delivery modes. At day 24, mice were sacrificed and dissected. The excised tumors were collected for evaluation of CD8 T cell infiltration using flow cytometry and immunofluorescence staining. For flow cytometry analysis, fresh tumors were cut into small pieces and digested using collagenase and DNase, followed by homogenizing in cold PBS to obtain single‐cell suspension. Afterward, the tumor cells were stained with BV510‐CD3 and eFluor 450‐CD8 antibodies to further detect the proportion of infiltrated CD8 T cells. Meanwhile, the excised tumors were immunofluorescence stained with OPAL multiplex IHC system and analyzed according to aforementioned procedures. CD8a primary antibodies (1:200) were detected with OPAL570 (1:100). Otherwise, the excised tibias were imaged via Quantum FX imaging system (PerkinElmer) and images were analyzed by Carestream MI SE software. Mice were considered dead when the tumor volume exceeded 1500 mm^3^ for 4T1 tumor model.

##### Postsurgical Recurrent Model and Treatment

Balb/c mice were preinoculated with luc‐4T1 (1 × 10^6^) cells at mammary fat pad (from day ‐7) and were performed surgery for tumor resection at day 0, leaving ≈1% residual tumor. The residual tumor was determined by comparing the bioluminescence F.I. of tumor before (day 1) and after tumor resection (day 0). Mice were then subcutaneously vaccinated with mono‐pulse CNVs (day 0), staggered‐pulse CNVs (days 0, 2, and 4), or gel‐confined CNVs (day 0). The total proteins amount of delivered CNVs was 660 µg in each delivery modes. Tumor burdens were observed at days 6, 18, and 24 using in vivo bioluminescence imaging system described before and were quantified as the average radiance (p s^−1^ cm^−2^ sr^−1^) using Living Image software. At day 24, mice were sacrificed and lungs were collected for H&E staining for lung metastasis analysis. Meanwhile, the relative tumor volume (RTV) of each tumor was obtained as the ratio of current volume to the initial volume, and then TGI was assessed by calculating the ratio of the mean RTV for CNVs treatment group to the mean RTV for the PBS group.

##### Lung Metastasis Model and Treatment

To establish lung metastasis model, Balb/c mice were intravenously infused with luc‐4T1 cells (1 × 10^5^) via tail vein at day 0 after vaccination with mono‐pulse CNVs (day ‐7), staggered‐pulse CNVs (days ‐7, ‐5, and ‐3), or gel‐confined CNVs (day ‐7). The total proteins amount of delivered CNVs was 660 µg in each delivery modes. In vivo bioluminescence imaging was conducted at days 5 and 18, and the bioluminescence signals of luc‐4T1 cells in lung region were quantified. At day 18, mice were also in vivo imaged via Quantum FX imaging system (PerkinElmer), and the lung images were processed by using Analyze software.

##### In Vivo Safety Evaluation

The safety evaluation in vivo was performed in four parts: body temperature and the body weight evolutions, levels of cytokines in serum, levels of important indicators in serum, and organ damages. After vaccinated with CNVs in three delivery modes, the body temperature and body weight of mice were recorded during the observing period. On day 7 postvaccination, mice were sacrificed and the blood samples and organs were collected. The serum levels of cytokines including IL‐6, TNF‐*α*, and IFN‐*γ* were detected by ELISA kits according to manufacturer's instruction. Hearts, livers, spleens, lungs, and kidneys were stained with H&E to evaluate organ dysfunction. For 4T1 tumor‐bearing mice in primary tumor models, blood samples were collected at day 27 and the serum levels of important indicators: alkaline phosphatase (ALP), blood urea nitrogen (BUN), aspartate transaminase (AST), and alanine aminotransferase (ALT) were analyzed using a Biochemical Autoanalyzer (TBA‐40, Toshiba).

##### E.G7 Primary Tumor Models and Treatment

To assess the therapeutic effects of M/S@Lipo nanovaccines, C57BL/6 mice were inoculated with E.G7 cells (5 × 10^5^) at axillary at day 0 and vaccinated with monopulse M/S@Lipo (day 3), staggered‐pulse M/S@Lipo (days 3, 5, and 7), or gel‐confined M/S@Lipo (day 3). The total delivered amount of SIINFEKL peptides was 200 µg in each delivery mode. Tumor volumes were recorded every 2–3 d as described before. At day 21, TGI calculation was conducted according to the aforementioned method. Mice were considered dead when the tumor volume exceeded 2500 mm^3^ for E.G7 tumor model.

##### Statistical Analysis

All data were presented as mean ± s.e.m. and were compared by means of an unpaired, two‐tailed Student's *t*‐test (two groups) or one‐way ANOVA with Tukey post‐hoc test (multiple groups). Log‐rank test was used for survival analysis. Sample sizes (*n*) were mentioned on each figure legend. All the statistical analyses were conducted by using GraphPad Prism software (version 8.0). Asterisks indicated significant differences (*P < 0.05, **P < 0.01, ***P < 0.001, and ****P < 0.0001). All animal studies were performed after randomization.

## Conflict of Interest

The authors declare no conflict of interest.

## Supporting information

Supporting Information
