# Supplementary material for: Choice of Nanovaccine Delivery Mode Has Profound Impacts on the Intralymph Node Spatiotemporal Distribution and Immunotherapy Efficacy
Source: Adv Sci (Weinh). 2020 Aug 15;7(19):2001108. doi: 10.1002/advs.202001108 (PMC7539204; doi:10.1002/advs.202001108)
Supplement: Supplementary file 1 — Supporting Information [file ADVS-7-2001108-s001.pdf]

© 2020 Wiley-VCH GmbH

ADVANCED SCIENCE Open Access

Supporting Information

for *Adv. Sci.*, DOI: 10.1002/advs.202001108

Choice of Nanovaccine Delivery Mode Has Profound Impacts on the Intralymph Node Spatiotemporal Distribution and Immunotherapy Efficacy

Jianghua Wang, Shuang Wang, Tong Ye, Feng Li, Xiaoyong Gao, Yan Wang, Peng Ye, Shuang Qing, Changlong Wang, Hua Yue, Jie Wu, Wei Wei, and Guanghui Ma**

Copyright WILEY-VCH Verlag GmbH & Co. KGaA, 69469 Weinheim, Germany, 2020.

Supporting Information

Choice of nanovaccine delivery mode has profound impacts on the intra-lymph node spatiotemporal distribution and immunotherapy efficacy

Jianghua Wang, Shuang Wang, Tong Ye, Feng Li, Xiaoyong Gao, Yan Wang, Peng Ye, Shuang Qing, Changlong Wang, Hua Yue, Jie Wu, Wei Wei*, and Guanghui Ma*

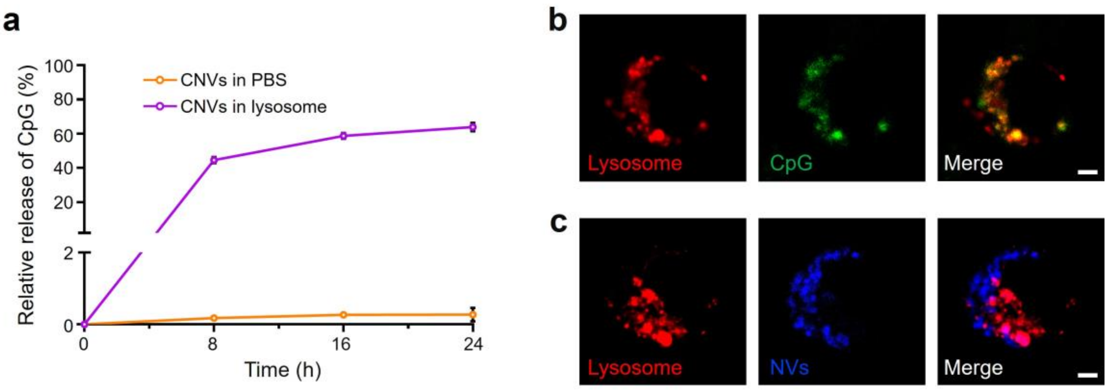

Figure S1. The release behavior of antigen and CpG in vitro. a) The release profile of fluorescein-CpG for CNVs under lysosomal inclusion and PBS in vitro. b,c) CLSM images for the localization of fluorescein-CpG (b, green) or DiD-labeled NVs (c, blue) with lysosomes (red) in DCs after 12 h treatment. Scale bar: 2 μm. The results showed that CNVs had the capacity of selectively releasing CpG in lysosomal environment, where CpG could react with TLR9 to promote DC activation and NVs could be utilized for antigen presentation. Data represent the mean ± s.e.m. (n = 3).

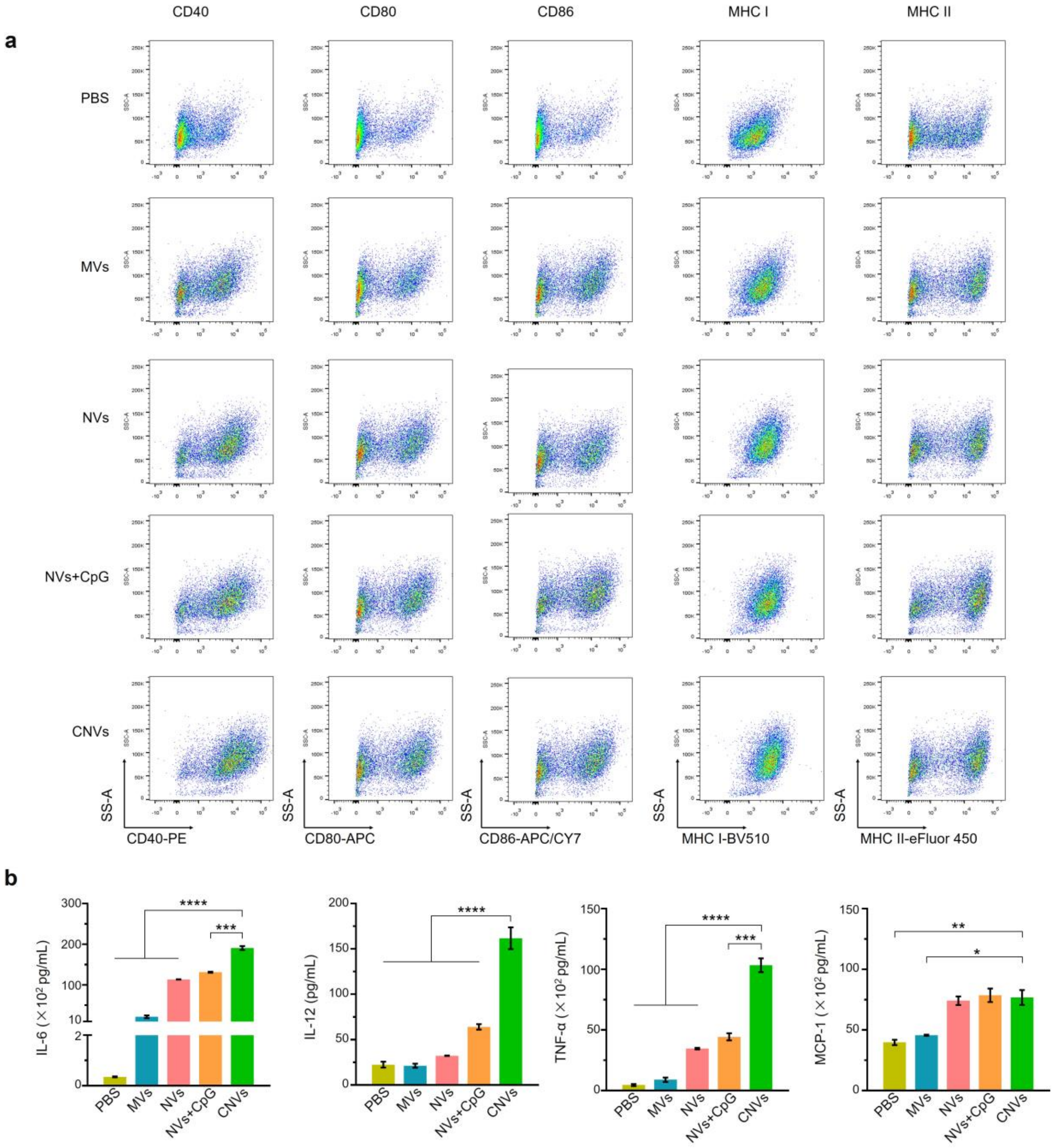

Figure S2. BMDCs activation induced by CNVs in vitro. a) Flow cytometry dot plots of costimulatory molecules (CD40, CD80, and CD86) and MHC molecules (MHC I and MHC II) on BMDCs (gating on 5×10^3 $CD11c^+$ cells) after 24 h incubation with MVs, NVs, NVs plus CpG, or CNVs. b) The released cytokines levels (IL-6, IL-12, TNF-α, and MCP-1) in the culture supernatants of BMDCs harvested from experiment in panel a. Collectively, these results indicated the immune activation ability of CNVs. Data represent the mean ± s.e.m. (n = 3). P-values were calculated via one-way ANOVA with Tukey post-hoc test. *P < 0.05, **P <0.01, ***P < 0. 001, ****P < 0.0001.

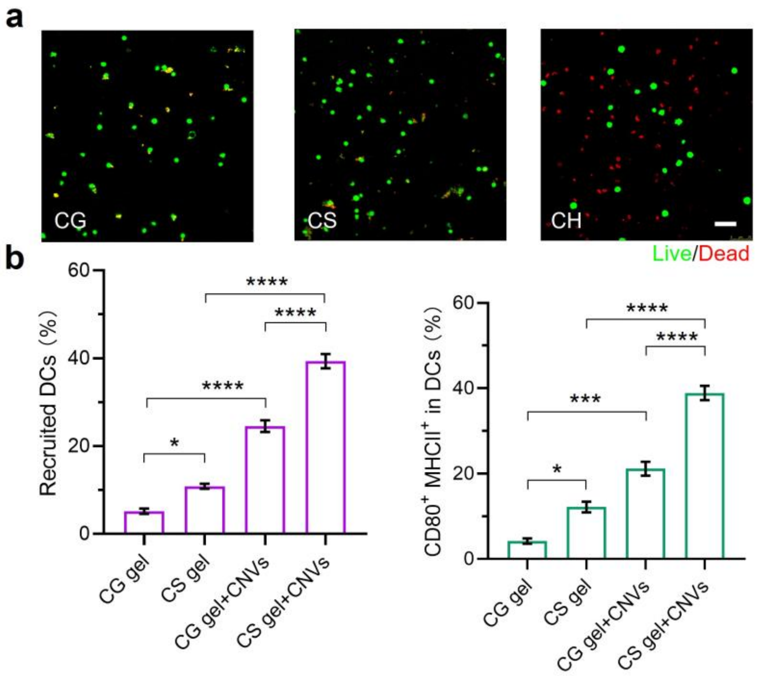

Figure S3. Comparation of chitosan glutamate (CG) gel, chitosan (CS) gel and chitosan hyamine (CH) gel on adjuvant effect and safety in vivo. a) Live/Dead stained images of isolated cells from the retrieved gel samples. Almost all cells in both CG and CS groups remained alive (green), while exposure of CH significantly induced a proportion of dead cells (red) due to its highly positive-charged nature. Scale bar: 25 μm. b) Proportions of the recruited ($CD11c^{+}$) and activated DCs ($CD80^{+}$ MHC II^{+}). CS gel significantly promoted the recruitment and activation of DCs rather than CG gel, indicating the potent adjuvant effect of CS and almost non-adjuvant property of CG. Once the CNVs were added, DC recruitment and activation were both further improved greatly, but still in increasing order of CG gel and CS gel. Data represent the mean ± s.e.m. (n = 3). P-values were calculated via one-way ANOVA with Tukey post-hoc test. *P <0.05, ***P <0.001, ****P <0.001.

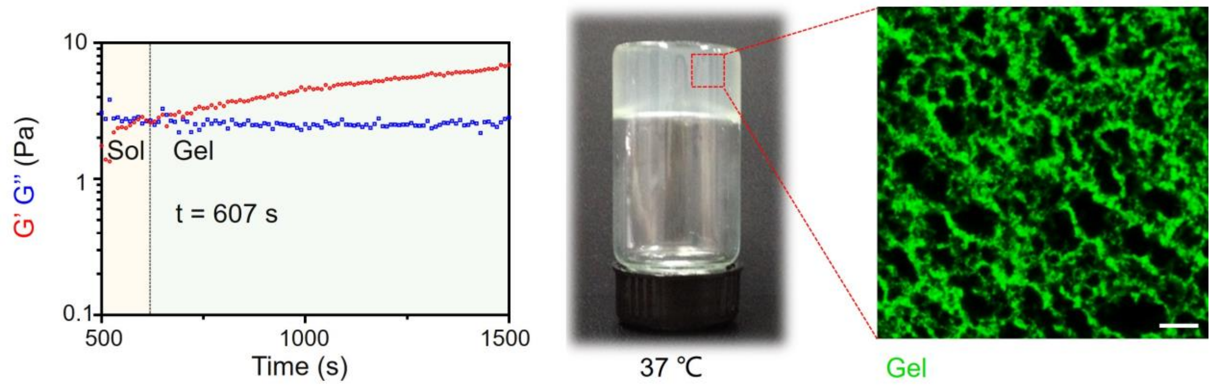

Figure S4. Thermosensitive property of CG hydrogel in vitro. The evolution of dynamic storage modulus (G') and loss modulus (G'') of chitosan hydrogel at 37 ℃ (left), and the intersection of G' and G'' line indicated the gelation time of hydrogel was 607 s (~10 minutes). Moreover, the photograph showed the gel formation of the chitosan hydrogel after incubation at 37 ℃ (middle), providing a porous network structure (green) to embed CNVs (right). Scale bar: 50 μm. All these results together demonstrated that the as-prepared CG hydrogel was thermosensitive allowing gelation at body temperature of animal.

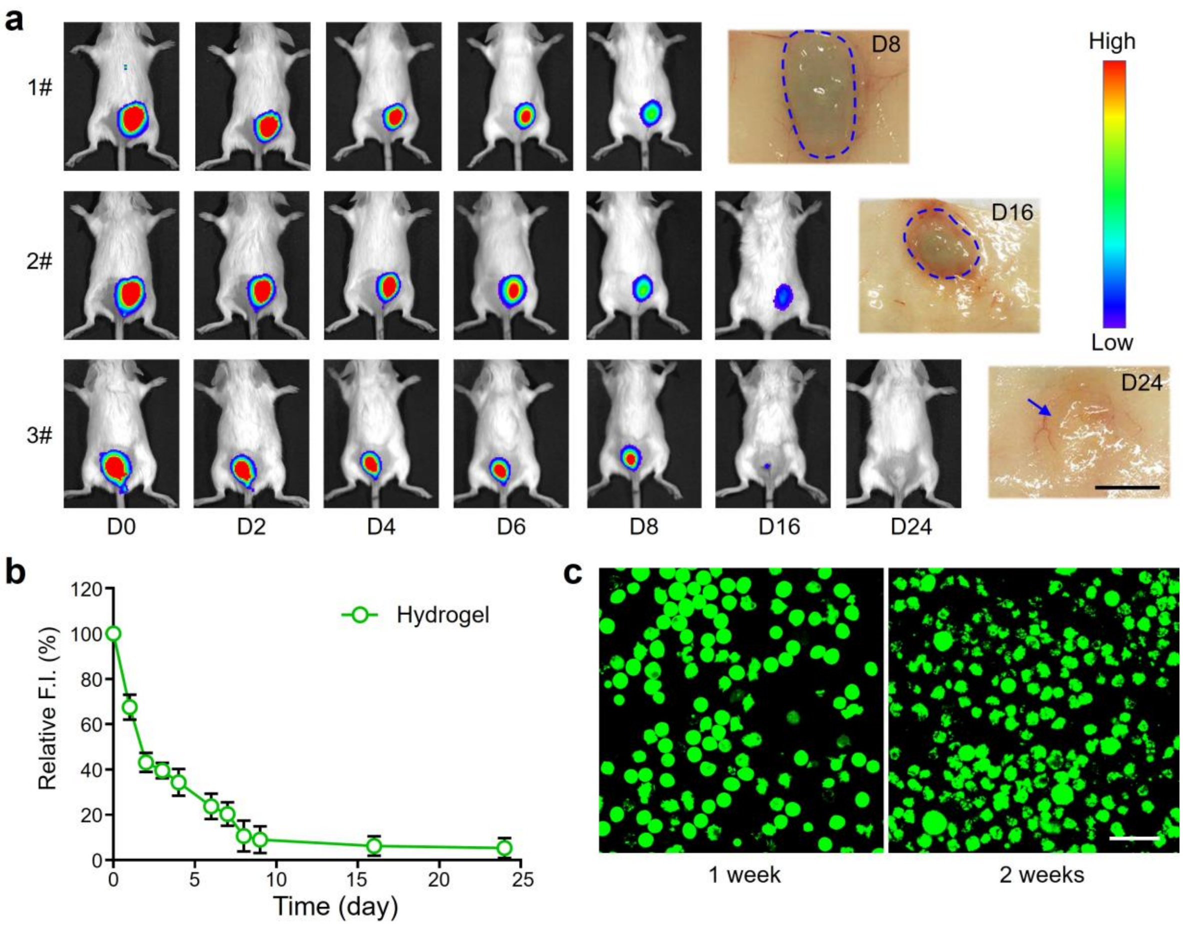

Figure S5. Biodegradability and biocompatibility of gel-confined CNVs in vivo. a) Representative in vivo images of three mice at different time points after *s.c.* injection with the mixture solution of cyanine 7 (Cy7)-labeled hydrogel and CNVs (left) and the photographs of excised tissue from the injection site of each mice at days 8, 16, and 24 post-injection respectively (right). Scale bar: 1 cm. b) Quantification of fluorescence intensity (F.I.) of Cy7-labeled hydrogel at different time points. The results showed that the fluorescent signal of hydrogel gradually decayed within 3 weeks, and there were no signs of skin damage during hydrogel degradation, indicating the biodegradability and biocompatibility of gel-confined CNVs injection. c) Live (green) / Dead (red) stained images of recruited cells isolated from retrieved gel samples from mice at 1 week or 2 weeks after injection with gel-confined CNVs. Scale bar: 25 μm. The result showed that almost all the recruited cells were viable, again confirming the good biocompatibility of gel-confined CNVs. Data represent the mean ± s.e.m. (n = 3).

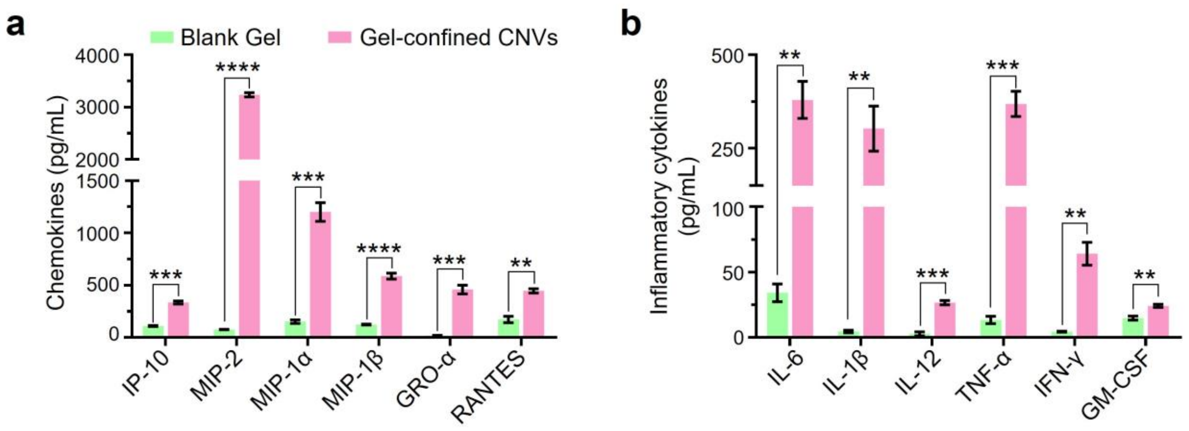

Figure S6. Local immune microenvironment induced by gel-confined CNVs at injection site. a,b) The levels of chemokines (a) and pro-inflammation cytokines (b) in lysates of the isolated cells from retrieved gel samples at day 3 after injection with blank gel or gel-confined CNVs. The significant upregulation of chemokines and pro-inflammation cytokines indicated the efficient recruitment and activation of DCs at injection site induced by gel-confined CNVs compared with blank gel. Data represent the mean ± s.e.m. (n = 3). P-values between two groups were calculated via unpaired Student's t-test. $^{**}P < 0.01$, $^{***}P < 0.001$, $^{****}P < 0.0001$.

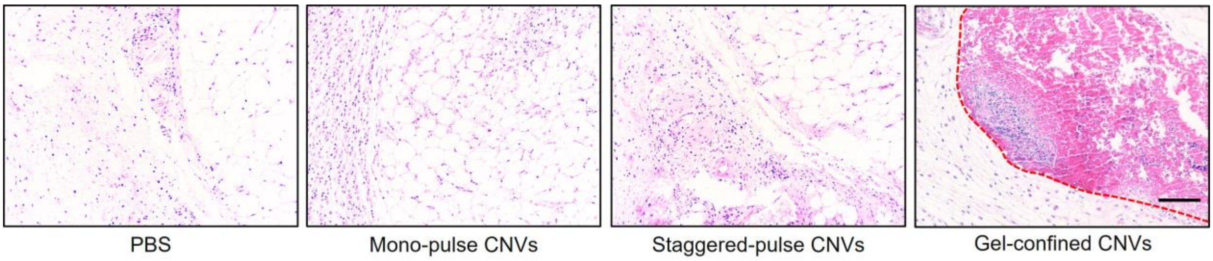

Figure S7. Recruitment behavior at the injection site after *s.c.* vaccination under three CNVs delivery modes. H&E stained micrographs of skin tissues at the injection site collected from mice of three CNVs delivery modes at 12 h post-injection. Scale bar: 100 μm. Compared with PBS group, almost no APCs recruitment were observed after mono-pulse CNVs and staggered-pulse CNVs vaccinations. On the contrary, vaccination with gel-confined CNVs rapidly triggered the APCs recruitment within 12 h, which could avoid the leaking of CNVs and maximize the supply for recruited APCs.

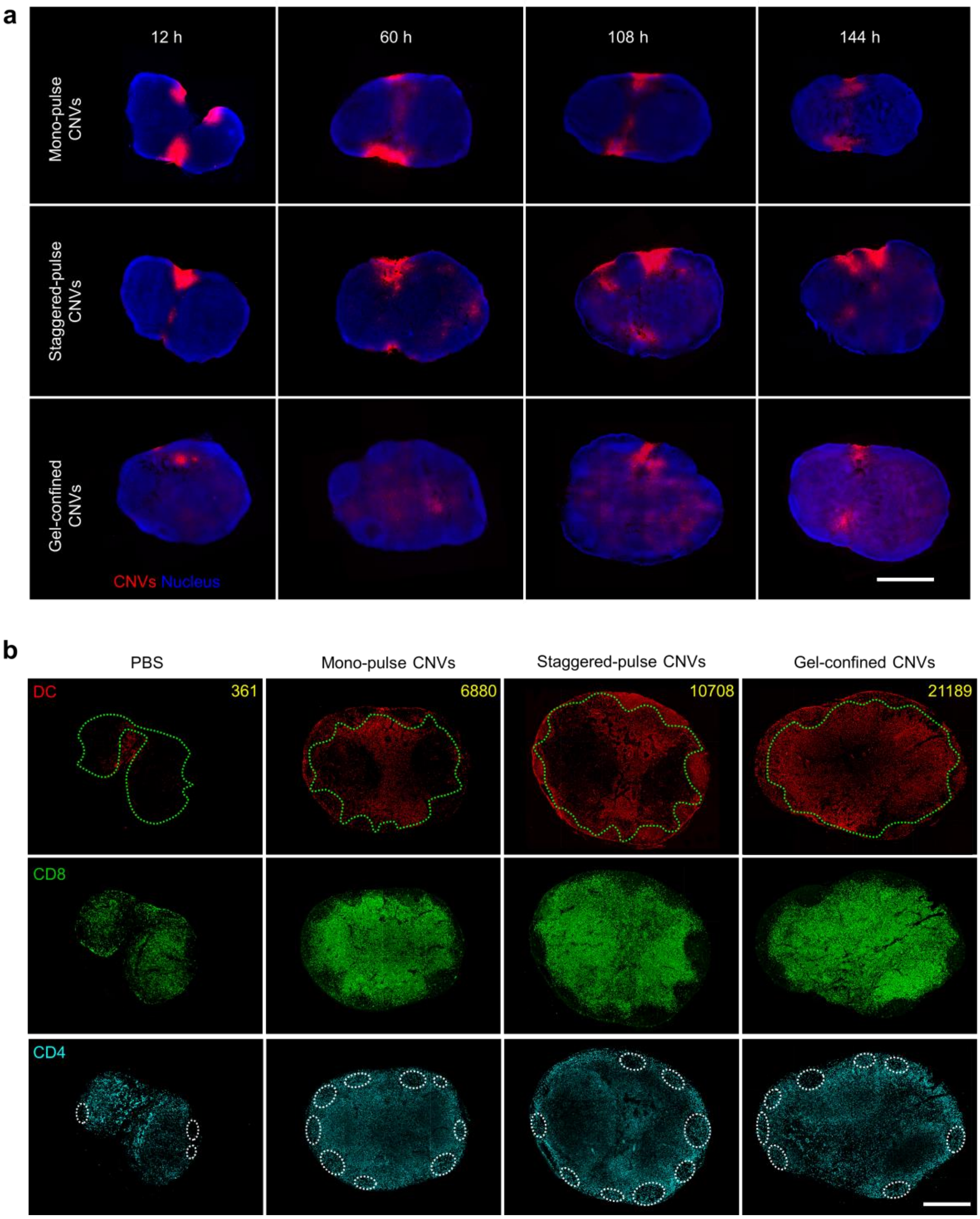

Figure S8. Intra-lymph node distribution of CNVs and immune cells after vaccination with three CNVs delivery modes. a) Intra-lymph node distribution of CNVs among three delivery modes at different time points. Scale bar: 2 mm. b) Multiple immunofluorescence analysis of immune cells in lymph nodes. DCs (red), CD8 T cells (green), and CD4 T cells (cyan). Scale bar: 1 mm. The number in the upper right corner indicated the quantitation of infiltrated DC in paracortex via Inform Image Analysis software (PerkinElmer).The result showed that all three CNVs delivery modes displayed distinct CNVs distribution patterns, and subsequently induced the infiltration of DCs into the paracortical T cell zone (top, green circle) and the migration of CD4 T cell into germinal centers (bottom, white circle), compared with control group. Deeper infiltration of DCs into was observed for gel-confined CNVs delivery mode, indicating the better potential T cells activation of gel-confined CNVs.

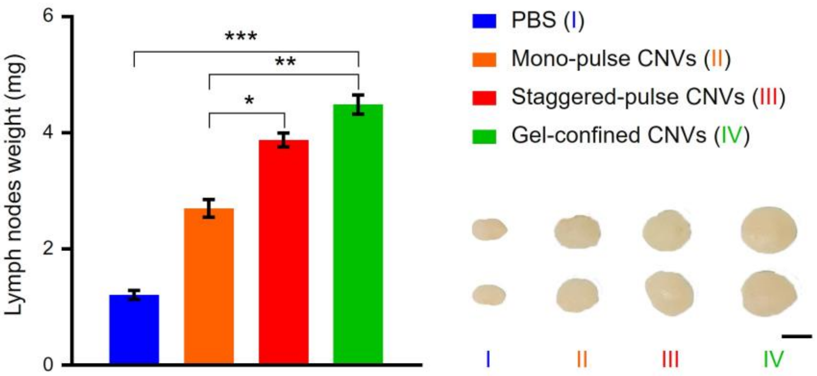

Figure S9. Immune response in lymph nodes stimulated by three CNVs delivery modes. Weight analysis of inguinal lymph nodes isolated from mice of three CNVs delivery mode groups at day 7 post-injection and the representative photographs of two inguinal lymph nodes resected from one mouse in each group. Scale bar: 2 mm. The results showed that there were substantial increases of lymph node weight under CNVs vaccination with three delivery modes, and the gel-confined CNVs delivery mode exhibited better potential for lymph node activation. Data represent the mean ± s.e.m. (n = 6). P-values were calculated via one-way ANOVA with Tukey post-hoc test. *$P < 0.05$, **$P < 0.01$, ***$P < 0.001$.

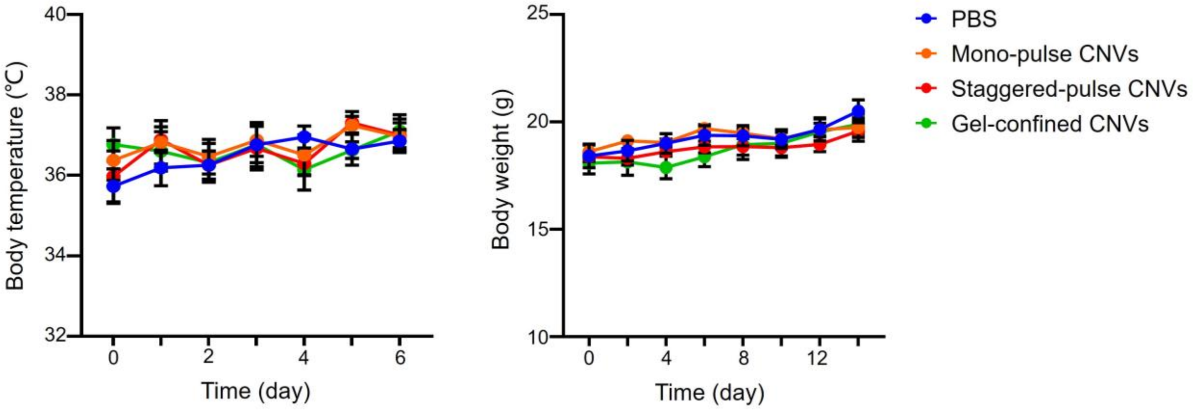

Figure S10. Body condition of mice after vaccination with three CNVs delivery modes. Body temperature and weight evolution for mice of the three CNVs delivery mode groups. The results showed no significant changes between healthy mice and CNVs treated mice. Data represent the mean ± s.e.m. (n = 6).

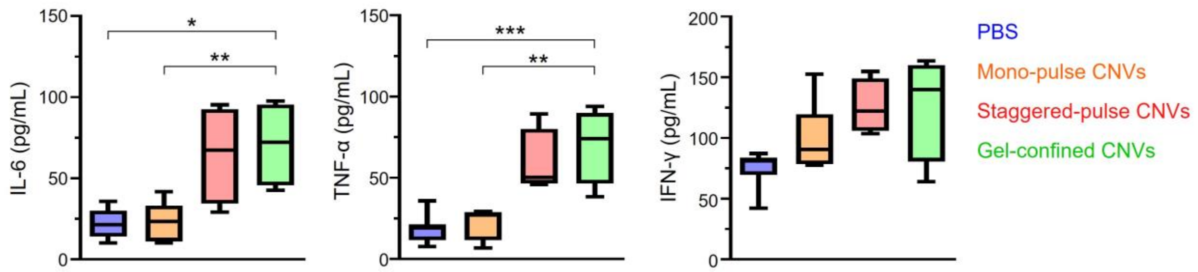

Figure S11. Systemic immune response induced by three CNVs delivery modes. Quantitation of cytokine-storm-related cytokines (IL-6, TNF-α, and IFN-γ) in serum samples from mice of the three CNVs delivery mode groups at day 7 post-injection. The results showed that the levels of all three cytokines elevated after vaccination in three delivery modes. However, such enhancements (< 5-fold increase) had no risk of cytokine storm, which usually induced over 100-fold upregulation of cytokines compared to healthy mice. Data represent the mean ± s.e.m. (n = 3). P-values were calculated via one-way ANOVA with Tukey post-hoc test. *P < 0.05, **P < 0.01, ***P < 0.001.

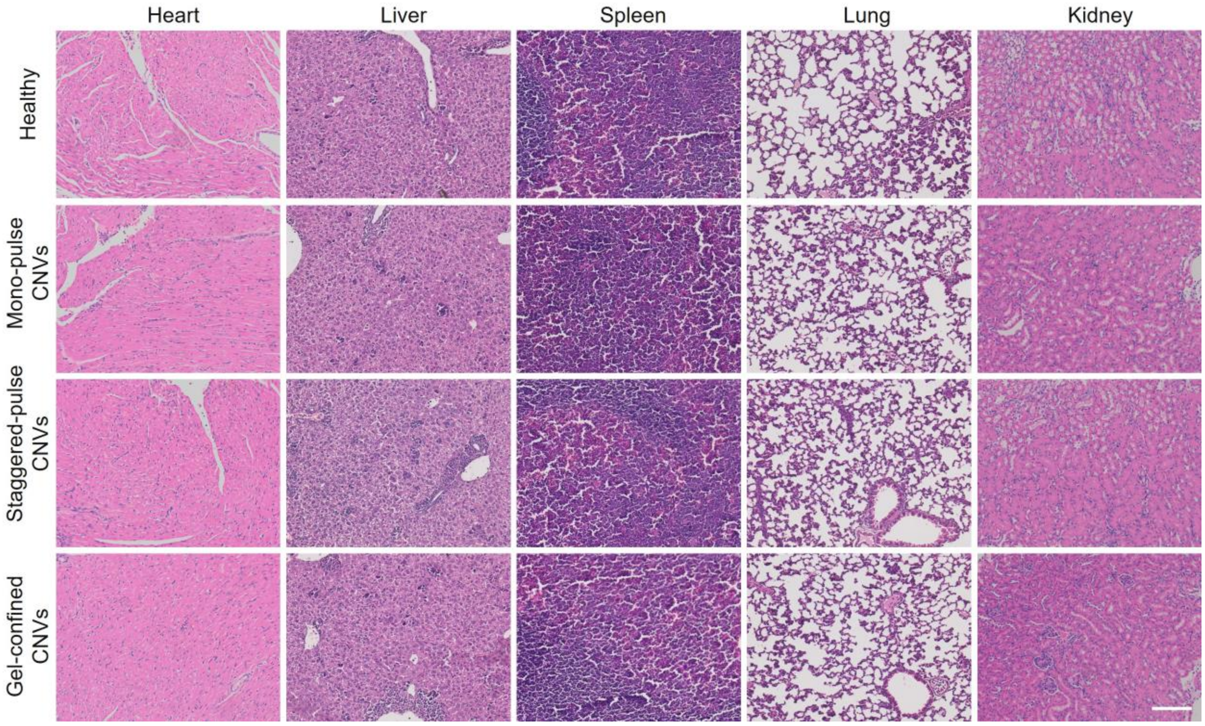

Figure S12. Biosafety of CNVs vaccination under three delivery modes. H&E stained micrographs of various organs collected from mice of the three CNVs delivery mode groups at day 7 post-vaccination, which showed no signals of organ damage, suggesting the biosafety of CNVs vaccination under three delivery modes. Scale bar: 100 μm.

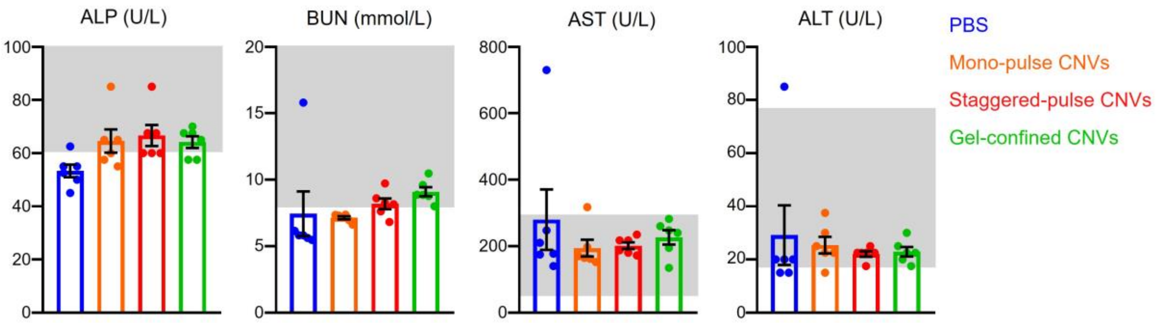

Figure S13. Health level of mice in prophylactic 4T1 primary tumor model. Serum biochemical values including alkaline phosphatase (ALP), blood urea nitrogen (BUN), aspartate aminotransferase (AST), and alanine aminotransferase (ALT) in 4T1 tumor bearing mice in prophylactic primary tumor model. The data showed that all indexes levels were in the range or closed to the data of healthy mice (gray area) after treatment with staggered-pulse CNVs and gel-confined CNVs, as compared to PBS control and mono-pulse CNVs treatment, indicating the good health condition of mice. Data represent the mean ± s.e.m. (n = 6).

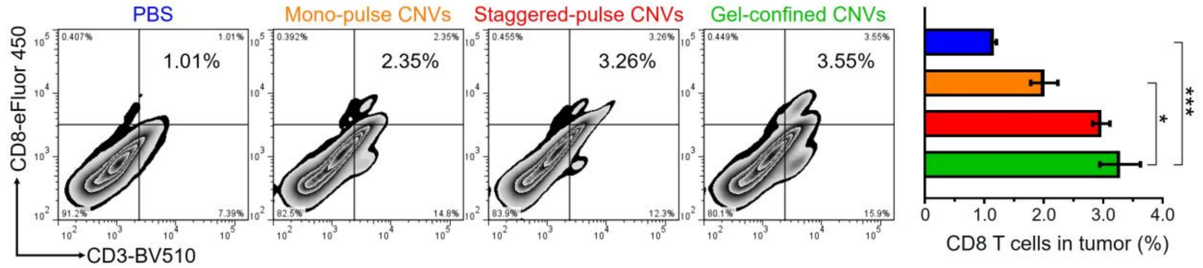

Figure S14. The infiltration of CD8 T cells in tumor via flow cytometry analysis. The results showed that the proportion of infiltrated CD8 T cells in tumor increased in the order of PBS, mono-pulse CNVs, staggered-pulse CNVs and gel-confined CNVs, which were consistent with the fluorescence images in Figure 4g. Data represent the mean ± s.e.m. (n = 3). P-values were calculated via one-way ANOVA with Tukey post-hoc test. *P < 0.05, ***P < 0.001.

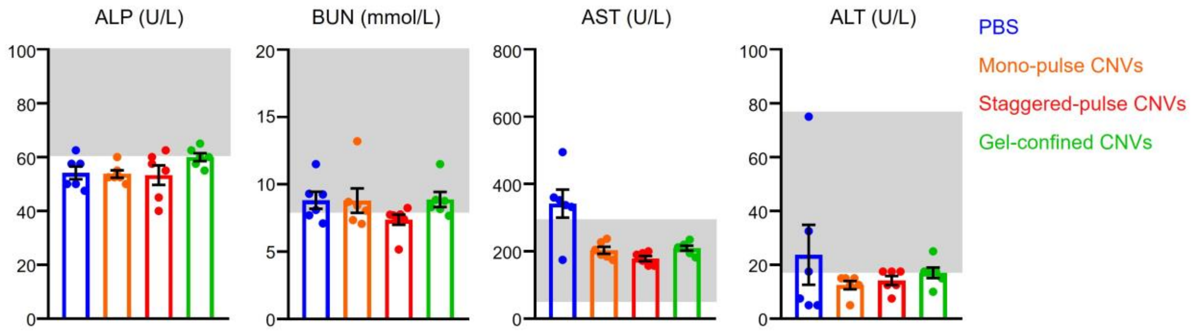

Figure S15. The health level of mice in therapeutic 4T1 primary tumor model. Serum biochemical values (ALP, BUN, AST, and ALT) of 4T1 tumor-bearing mice in therapeutic primary tumor model. The data showed that the indexes levels were in the range or closed to the data of healthy mice (gray area) after treatments with CNVs in three delivery modes, compared to PBS control, indicating the amelioration of health condition for mice. Data represent the mean ± s.e.m. (n = 6).

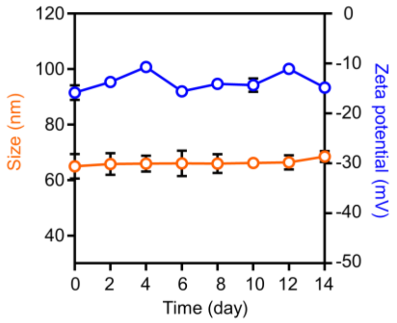

Figure S16. Stability of M/S@Lipo stored in PBS over a two-week period. There were no changes of size and zeta potential for M/S@Lipo during storage in PBS at 4 °C for two weeks, confirming the good stability of liposomes. Data represent the mean ± s.e.m. (n = 3).
